# Acceptability and Utility of an Open-Access, Online Single-Session Intervention Platform for Adolescent Mental Health

**DOI:** 10.2196/20513

**Published:** 2020-06-30

**Authors:** Jessica Lee Schleider, Mallory Dobias, Jenna Sung, Emma Mumper, Michael C Mullarkey

**Affiliations:** 1 Department of Psychology Stony Brook University Stony Brook, NY United States

**Keywords:** internet intervention, online interventions, youth, mental health, adolescent, depression, single-session intervention, intervention

## Abstract

**Background:**

Many youths with mental health needs are unable to access care. Single-session interventions (SSIs) have helped reduce youth psychopathology across multiple trials, promising to broaden access to effective, low-intensity supports. Online, self-guided SSIs may be uniquely scalable, particularly if they are freely available for as-needed use. However, the acceptability of online SSI and their efficacy have remained unexamined outside of controlled trials, and their practical utility is poorly understood.

**Objective:**

We evaluated the perceived acceptability and proximal effects of Project YES (Youth Empowerment & Support), an open-access platform offering three online SSIs for youth internalizing distress.

**Methods:**

After selecting one of three SSIs to complete, participants (ages 11-17 years) reported pre- and post-SSI levels of clinically relevant outcomes that SSIs may target (eg, hopelessness, self-hate) and perceived SSI acceptability. User-pattern variables, demographics, and depressive symptoms were collected to characterize youths engaging with YES.

**Results:**

From September 2019 through March 2020, 694 youths accessed YES, 539 began, and 187 completed a 30-minute, self-guided SSI. SSI completers reported clinically elevated depressive symptoms, on average, and were diverse on several dimensions (53.75% non-white; 78.10% female; 43.23% sexual minorities). Regardless of SSI selection, completers reported pre- to post-program reductions in hopelessness (*d*_av_=0.53; *d*_z_=0.71), self-hate (*d*_av_=0.32; *d*_z_=0.61), perceived control (*d*_av_=0.60; *d*_z_=0.72) and agency (*d*_av_=0.39; *d*_z_=0.50). Youths rated all SSIs as acceptable (eg, enjoyable, likely to help peers).

**Conclusions:**

Results support the perceived acceptability and utility of open-access, free-of-charge SSIs for youth experiencing internalizing distress.

**Trial Registration:**

Open Science Framework; osf.io/e52p3

## Introduction

The persistent unmet need for youth mental health services, combined with the ubiquity of internet-equipped devices, has fueled a surge of interest in digital mental health interventions. Despite decades of progress in the identification of evidence-supported psychosocial treatments, myriad barriers—both systemic (high costs, transportation challenges) and internal (stigma, preferences for self-help)—prevent up to 80% of youth from accessing needed care [1,2]. The notion of low-cost, self-guided digital interventions thus appeals to researchers, providers, and clients alike. Indeed, mental healthcare apps are downloaded by millions of users annually [3,4], and a vast majority of adolescents in the United States have access to internet-equipped smartphones (95%) and computers (88%) [5]. These realities create opportunities for youth with no accessible alternatives to engage with digital supports.

Randomized trials suggest the promise of internet- and smartphone-based youth mental health tools [6-10]. However, formal clinical trial outcomes may not reflect the utility or usability of digital supports in naturalistic contexts. First, few evidence-based digital interventions developed for clinical trials are publicly accessible [11], and those that are accessible attract far fewer users than commercially popular but untested alternatives (eg, Headspace) [12]. Indeed, the most frequently downloaded mental health apps include *few* to *no* treatment elements found in empirically supported psychotherapies [13,14]. Second, digital intervention attrition rates differ markedly in clinical trials and “real-world” contexts. In a review of digital self-help tools, including apps and internet-based programs for depression and anxiety, completion rates for programs in clinical trials were 44%-99%. In contrast, completion rates for the same programs in naturalistic, non-research settings—where participants received no incentives for program engagement—were 1%-28% [15].

Overall, daily usage rates for mental health apps hovers at just 4% (based on rates of opening a previously downloaded program) [7]. Reasons for low engagement in youth-directed mental health apps include lack of perceived program value, limits on program accessibility (eg, within-program fees, limits on 24/7 access), and poor user experience (eg, high demands on users’ time) [4,16]. Thus, a need exists for digital mental health supports that (1) include evidence-based content, established in well-controlled trials; (2) show acceptability in naturalistic contexts (ie, outside of formal trials); (3) minimize demands on users’ time, given the high likelihood of accessing a digital intervention only once; and (4) are free of access barriers, such as program costs.

Single-session interventions (SSIs)—structured interventions, including *one* component of empirically supported treatments and requiring *one* encounter with a provider or program—could dramatically improve the acceptability and accessibility of digital mental health tools. Overall, SSI effects on youth psychopathology are slightly smaller than those observed for youth treatments lasting an average of 16 sessions (*g*=0.32 for single-session interventions [17], versus *g*=0.46 for multi-session interventions [17,18]. However, their brevity and flexible format magnify their potential public health impact [19]. Additionally, across placebo-controlled trials, self-administered online SSIs have reduced youth depression across 4-9 months in high-symptom and community youth samples [20-22]. These findings suggest the untapped potential of online SSIs to improve youth access to effective support.

In principle, online SSIs might overcome many challenges typical of traditional digital mental health tools. They are completable within one sitting, minimizing engagement burdens on users, and many are cost-free, publicly accessible, and include evidence-based intervention components. However, there have been no formal evaluations of the acceptability of online SSIs outside of controlled clinical trials. To gauge the promise and challenges to realizing the potential of online SSIs to benefit youth mental health, we must systematically evaluate usage patterns, perceived acceptability, and the short-term effects of online SSIs in naturalistic contexts.

Toward this goal, we conducted an open-access program evaluation of online SSIs for adolescent mental health, Project YES (Youth Empowerment & Support) [23], to evaluate the acceptability and initial effects of three free-of-charge, publicly available web-based SSIs for adolescent internalizing distress. In line with SSI design recommendations, each of these three SSIs was intended to target and strengthen *adaptive beliefs* about the self and the world that have been shown to predict youth internalizing distress.

The first SSI, Project Personality, teaches how and why people’s traits—including their symptoms—are malleable rather than fixed [20]. Project Personality aims to strengthen adolescents’ *perceived control* over their behavior and emotions while *reducing hopelessness* regarding a positive future change. Low perceived control and elevated hopelessness consistently predict the course and severity of youth internalizing problems [24-28]. Further, both perceived control and hopelessness are modifiable via brief intervention, making them ideal proximal SSI targets. In a recent placebo-controlled trial, Project Personality significantly improved perceived control over emotions and behavior in adolescents characterized as high-symptom [15], and larger proximal increases in perceived control following Project Personality predicted greater reductions in internalizing symptoms over time [29]. Thus, in the context of Project YES, we assessed Project Personality’s link with improvements in perceived control and hopelessness.

The second SSI, Project CARE, teaches how and why one can work towards acting with self-compassion in order to reduce self-hate systematically [30]. Self-hate and closely related constructs (ie, self-criticism) are linked with current and future internalizing problems in both youths and adults [31-35]. Preliminary evidence also suggests negative perceptions of the self, including self-hate, are *modifiable* through self-administered interventions as brief as 5-11 minutes [36-39]. Thus, within Project YES, we assessed Project CARE’s link with improvements in self-hate.

The third SSI, the ABC Project, is modeled after behavioral activation interventions targeting adolescent depression, which teach that engaging in valued activities can powerfully shape one’s mood [30,40]. The construct of *agency—*one’s self-perceived ability to initiate and work towards goals—is central to mechanisms thought to underlie behavioral activation for youth depression. Specifically, “agency” references an individual’s perceived capacity to (a) set behavioral goals, and (b) maintain or generate motivation to move toward those goals [41]. Behavioral activation for adolescent depression—including a one-session, therapist-delivered program [42]—has produced marked improvements in adolescents’ perceived agency and depression symptom severity, relative to control conditions [43-45] Thus, within Project YES, we assessed the ABC Project’s link with proximal improvements in agency.

Each 30-minute program was created per a routinely employed SSI design framework, informed by basic social-psychological research and detailed elsewhere [19], wherein youths (1) learn brain science that normalizes each program’s core concept; (2) are treated as “experts” and invited to help researchers learn about youths’ perspectives and challenges; (3) synthesize the program’s ideas using their own words, while offering advice to peers facing difficulties; (4) hear stories from peers who used the program’s content to overcome setbacks. Regardless of SSI selection, youths taking part in YES are invited to offer their “best, anonymous coping advice” to other teens coping with depression or anxiety; each receives the option to share this advice to a public “YES advice center.” This feature builds on research suggesting that helping or supporting peers can benefit an individual’s sense of self-efficacy, motivation, and psychosocial functioning [46-48].

All YES participants may complete anonymous pre- and post-program assessments of proximal outcomes that may be altered by SSIs (per prior recommendations for evaluating SSI effects; [19]) along with feedback on program acceptability. Using pre-SSI, post-SSI, and user-pattern data collected from YES participants ages 11-17 over 7 months, we investigated the acceptability of YES SSIs among youths, including completion rates and perceptions of each SSI’s utility. We also assessed the *immediate effects* of YES SSIs on interrelated and clinically relevant outcomes they are designed to target, each of which relates to youth internalizing problems (agency, perceived control, hopelessness, and self-hate, assessed using both standardized and study-specific metrics). Specifically, we examined usage-pattern variables (eg, SSI selection, SSI completion rates), user characteristics (age-range, sex, gender identity, race/ethnicity, depressive symptoms), and acceptability metrics to gauge *which youths* use YES; *how* and *why* they engage with it; and whether they view YES as valuable, helpful, and user-friendly. We also examined whether proximal, clinically relevant outcomes significantly improved from pre- to post-SSI, both across all YES programs and within each program. This nonexperimental, observational project represents a critical step in understanding the promise of online mental health SSIs, and the challenges to realizing that promise.

## Methods

### Participants

Participants were youths ages 11-17 who took part in YES between September 19, 2019, and March 10, 2020. Participants learned about YES through various channels, including unpaid posts to social media websites (Reddit, Twitter), Instagram advertisements (total costs <$500), or an article published in *Vox* highlighting the authors’ research [49]. Social media posts included invitations to participate in an anonymous program evaluation wherein “teens can learn new ways of dealing with stress and help others do the same.” Posts linked to the public YES website [23], which includes additional information and a participation link. YES is completable from any internet-equipped device. Before YES was initiated, all procedures were reviewed and deemed “exempt” (as a program evaluation) by the University’s Institutional Review Board.

Because YES is anonymous and publicly accessible, no inclusion or exclusion criteria are used (as none are enforceable). Thus, adults and children aged 10 or younger may choose to participate, although the YES website states that programs are meant for youths under age 18—primarily, pre-adolescent and adolescent youth. Within YES, participants are required to report their age range and whether they are adults (>18). Using these data, we limited analyses to our population of interest (youths under age 18).

### Procedures

YES is a non-randomized, exploratory program evaluation; procedures and analyses were preregistered on Open Science Framework [50]. In order to maintain anonymity and minimize access barriers (eg, discomfort disclosing psychological distress, as parents are often unaware of their adolescents’ depressive symptoms, including suicidal and morbid ideation, in up to 80% of cases [51,52]), parent permission is not required to participate (waived by the University IRB). After clicking the YES participation link and advancing past a “Project Information” page where agreement to participate is indicated, participants provide non-identifying demographic information; select one of three SSIs to complete; and may then complete pre-SSI questionnaires, the SSI, and post-SSI questionnaires, designed to gauge the SSI’s acceptability and short-term effects. Lastly, participants are invited to anonymously share their “best advice for others dealing with depression, anxiety, or stress.” They may elect or decline to share their advice in the YES “Advice Center” for others to see.

### Procedural Deviations

There were three minor deviations from the procedures described in our preregistration that warrant mention. First, to minimize participation burdens for those completing pre- and post-SSI questionnaires in project YES, we decided approximately one month into data collection to use only the 3-item “Agency” subscale of the State Hope Scale as opposed to the full 6-item scale; we also used a shortened 3-item version of the Self-Hatred Scale; these three items were formally selected using confirmatory factor analysis in a separate sample of N=246 adolescents from the same age group (see Multimedia Appendix 1). Second, the Self-Hatred Scale used in this study included 6 response options, not 7, as noted in the preregistration, although the anchors are the same as in the initially validated version and as noted in the preregistration. Lastly, we had initially preregistered an exploratory logistic regression to examine whether YES participants’ demographic characteristics or symptom levels predicted odds of SSI completion or choice of SSI. Due to high multicollinearity among preregistered predictors (eg, biological sex and gender identity; Variance Inflation Factor >10), results of this test could not be responsibly interpreted; thus, we do not report them in this manuscript. An alternative approach to conducting these analyses was applied, and results are reported in Multimedia Appendix 1.

### Interventions

Each SSI within Project YES is designed according to four primary elements proposed by Schleider and colleagues [19] for self-guided youth mental health SSIs. These elements were drawn from basic social-psychological research, along with qualities common among SSIs that have demonstrated efficacy [19,22,42,53,54]. Each YES SSI (1) uses brain science-based explanation to enhance content credibility; (2) empowers youth to take on an ‘expert’ or ‘helper’ role throughout the program; (3) includes guided writing assignments, often termed “saying-is-believing” or “self-persuasion” exercises, to enhance content internalization and generalization; and (4) includes testimonials from trusted others, such as older peers or scientific experts. A detailed description of these four principles of SSI design is provided elsewhere [19].

Materials for all SSIs in Project YES are publicly available via the Open Science Framework (Project Personality [55], Project CARE [30], and The ABC Project: [40]). All three are 30-minute, self-administered programs. All materials viewed by youths who take part in Project YES are publicly viewable on the Project YES website [23].

#### Project Personality

This SSI includes an introduction to the brain and a lesson on neuroplasticity; testimonials from older youths who describe their views that traits are malleable; stories by older youths, describing times when they used “growth mindsets” to persevere during social/emotional setbacks; study summaries noting how/why personality can change; and an exercise in which youths write notes to younger students, using scientific information to explain people’s capacity for change. Project Personality has demonstrated effectiveness in several trials [20,21,56].

#### Project CARE

This SSI includes: an introduction to the science behind why adolescents might think disliking themselves is necessary for success and thus fear self-compassion; scientific evidence and testimonials from other teens that being self-compassionate predicts being more successful socially and academically; evidence-based tips for overcoming common, fear of self-compassion based obstacles to self-compassion in day to day life; and an exercise in which youths write notes to younger students, using scientific information to explain the benefits of using self-kindness.

#### The ABC Project

This SSI draws from components of behavioral activation. The ABC Project introduces the concept that engaging in value-based activities that build pleasure and accomplishment can combat a sad mood and low self-esteem. It includes psychoeducation about depression, including how behavior shapes feelings and thoughts, walks participants through a life values assessment, where they identify key areas from which they draw enjoyment and meaning, and supports participants in creating an activity hierarchy, where they identify and personalize 3 activities to target for change. Lastly, the ABC Project includes an exercise in which youths write about benefits that might result from engaging in each activity, identify obstacles that might keep them from doing the activities and a strategy for overcoming identified obstacles.

### Measures

#### Demographics

Participants selected their age bracket (provided in ranges to maintain anonymity: 11 to 13, 14 to 16, 17 to <18), biological sex, gender identity, sexual orientation, and race/ethnicity.

#### Mood and Feelings Questionnaire–Short

The Mood and Feelings Questionnaire—Short (SMFQ) is a valid, reliable screening tool for depression in youth [57]. Pre-SSI, participants rated their agreement with 13 statements (eg, “I felt lonely”; “I felt miserable or unhappy”; “I felt I was no good anymore”) reflecting thoughts and feelings in the past two weeks on a 3-point Likert scale (0=“not true”; 2=“true”). Internal consistency was α=.92. Note that the SMFQ does not measure suicidal ideation or suicidality, which are not assessed in the context of Project YES.

#### State Hope Scale

The State Hope Scale [58] is a 6-item self-report scale designed to evaluate hope in youth, including two reliable subscales: agency and pathways. The “agency” subscale measures perceived ability to generate plans and work towards one’s goals (eg, “I can think of many ways to reach my current goals”); the “pathways” subscale reflects perceived success in meeting those goals (eg, “At this time, I am meeting the goals I have set for myself”). In Project YES, we expected possible shifts in *agency* scores, but not in *pathways* scores, as participants would not have had opportunities to pursue goals in a new way from pre- to immediately post-SSI. Thus, we indexed hope using the 3-item agency subscale of the State Hope Scale. Immediately pre- and post-intervention, participants rated each of 3 statements to reflect how they felt about themselves right now on an 8-point Likert scale (0=“definitely false”; 8=“definitely true”). Internal consistency was α=.74 and α=.82 at pre- and post-SSI, respectively.

#### Beck Hopelessness Scale-4

The Beck Hopelessness Scale-4 (BHS-4) [59] is a reliable, shortened version of the 20-item scale used to measure hopelessness in youth [60]. Immediately pre- and post-intervention, participants rated each of 4 statements to indicate their sense of hopelessness “right now, in this moment” on a 4-point Likert scale (0=“absolutely disagree”; 3=“absolutely agree”). Internal consistency was α=.85 and α=.89 at pre- and post-SSI, respectively.

#### Self-Hate Scale

The Self Hate Scale [61] is a reliable, 7-item measure designed to measure feelings of self-hatred. A shortened, 3-item version of the original scale was adapted for this study. Items were formally selected using confirmatory factor analysis in an initial sample of N=246 adolescents from the same age group (ages 11-17; see Multimedia Appendix 1). Immediately pre- and post-intervention, participants rated how true each of 3 statements was for them right now (“I hate myself,” “I feel disgusted when I think about myself,” “I feel ashamed of myself”) on a 6-point Likert scale (1=“not at all true for me”; 6=“very true for me”). Internal consistency was α=.92 and α=.94 at pre- and post-SSI, respectively.

#### Perceived Control

A single-item measure of adolescent perceived control was developed for this study. Immediately pre- and post-intervention, participants rated agreement with the statement, “right now, I feel like things are out of my control” from 0-10 (“not at all” to “a lot”).

#### Perceived Change in Hopelessness and Problem-Solving

Two questions, developed for use in the present study based on previously established guidelines for assessing subjectively perceived change following an intervention [62], evaluated participants’ perceived change in hopelessness and ability to solve problems. Immediately post-intervention, these questions asked, “to what extent are you feeling hopeless right now?” and, “to what extent are you able to solve the problems facing you right now?”, when “compared to before doing this activity.” Perceived change in hopelessness and problem-solving ability were both rated on a 5-point Likert scale (“much more hopeless” to “a lot less hopeless”; “much less able to solve problems” to “a lot more able to solve problems”). These measures were created based on established methods used to calculate the “smallest effect size of interest,” or the smallest possible effect size associated with a detectable, subjective change within individuals [62].

#### Program Feedback Scale

The Program Feedback Scale (PFS) [63], which is routinely used to evaluate acceptability and user perceptions of SSIs [64-67], asks participants to rate agreement with seven statements indicating perceived acceptability and feasibility of their selected SSI (eg, “I enjoyed the program”) on a 5-point Likert scale (1=“really disagree”; 5=“totally agree”). The scale was adapted from existing, validated acceptability assessments of digital interventions; adaptations from existing scales were necessary in order to exclude items that are inapplicable to web-based SSIs (eg, items referencing frequency of use or interest in continuing to revisit the program). The PFS also assessed participants’ open-response feedback. PFS item scores may be evaluated individually or via a mean-score across items. Internal consistency across PFS items was α=.88, but mean responses to *each* PFS items were considered independently in this study to inform understandings of acceptability in specific domains (eg, enjoyability, ease of use, ease of understanding).

### Analytic Plan

#### Sample Characterization and Usage Patterns

To assess YES usage patterns, we identified numbers of youths who began YES; selected, started, and completed an SSI; which SSI youths selected; and demographics, symptom levels, and hopelessness, self-hate, and for youths who selected, started, and completed an SSI. For youths who completed an SSI and the PFS, we computed overall and item-level means to assess the acceptability and feasibility of each SSI. Per our preregistration, mean scores of >3 on any PFS item indicates item endorsement (ie, positive feedback/adequate acceptability); a mean *overall* score of >3, across all items, reflects overall perceived SSI acceptability. Using the sub-sample of SSI completers, descriptive statistics for pre-to-post SSI “perceived change” items were computed. Mean ratings >0 on either item indicated an overall, subjectively detectable pre-to-post SSI change on that dimension (*hopelessness* or *problem-solving ability*).

#### SSI Effects on Proximal Outcomes

Within-group effect sizes (Cohen *d*, including 95% confidence intervals) were computed reflecting the change in pre-SSI to post-SSI levels of each immediate post-SSI outcome variable (hopelessness, self-hate, agency), both across and within SSIs. Because there are multiple approaches to computing Cohen *d* for within-subject designs [68], we report both *d*_av_ (1) and *d*_z_ (2) to maximize transparency:

Cohen *d_av_*=*M*_diff_/((*SD*_1_+*SD*_2_)/2) (**1**)

Cohen *d_z_*=M_diff_/√(∑((*x*_diff_–*M*_diff_)^2^/(*N*-1)) (**2**)

When the correlation between pre- and post-intervention outcome assessments is *r*>0.5, *d*_z_ is larger than *d*_av_; when *r*<0.5, Cohen *d*_z_ is smaller than Cohen *d*_av_. Whereas *d*_z_ accounts for within-subject correlations between pre- and post-program measures, *d*_av_ does not. Here, conclusions were drawn based on patterns of effect sizes across both approaches to computing Cohen *d*.

All available data were used for each test described above. Missing data rates are reported but not imputed, as usage patterns (including attrition) were of direct empirical interest. Anonymized data and code for all analyses are available via the Open Science Framework [50].

## Results

### Sample and Usage Patterns in YES

From September 2019 to March 2020, 694 youths accessed Project YES, of whom 612 selected, 539 began, and 187 finished a 30-minute SSI, for an overall completion rate of 34.32% among those who began an intervention (see Tables 1 and 2 for details). Those who selected, began, and completed an SSI reported clinically elevated depressive symptoms, on average (per an SMFQ cut-off score of 11 for early adolescents [69], established via comparisons with diagnostic interviews and validated depression screening measures), and were diverse on several dimensions (across all youths who accessed YES: 53.28% non-white, N=373; 78.98% biologically female, N=542; 50.13% identified as non-heterosexual, N=399; see Table 1 for details). Among those who selected an SSI, 43.30% (N=265) chose Project Personality (30.67% completion rate among those who began the SSI, N=74), 19.45% (N=119) chose Project CARE (37.37% completion rate among those who began the SSI, N=38), and 37.25% (N=228) chose the ABC Project (37.13% completion rate among those who began the SSI, N=75). Completion rates did not differ significantly from one another. Among youths who began an SSI, users ages 11-13 completed their chosen programs at higher rates than older users (57.75% of 11-13 year-olds, N=41; 29.45% of 14-16 year-olds, N=97; and 33.09% of 17 to <18 year-olds, N=46). Biological sex (male versus female versus intersex), sexual orientation (non-heterosexual versus heterosexual), racial/ethnic identity, and depressive symptom severity (total SMFQ score) were not associated with odds of SSI completion.

**Table 1:** Full sample demographics by SSI completion status.

Demographic characteristic		Total Sample, mean (SD) or N (%)	Selected an SSI, mean (SD) or N (%)	Started an SSI, mean (SD) or N (%)	Completed an SSI, mean (SD) or N (%)
N		694	612	539	187
SMFQ^a^		17.09 (6.41)	17.09 (6.41)	17.09 (6.43)	16.51 (6.34)
**Age**					
	<10	6 (0.86%)	5 (0.82%)	3 (0.56%)	3 (1.60%)
	11 to <13	93 (13.40%)	80 (13.07%)	71 (13.17%)	41 (21.93%)
	14 to <16	416 (59.94%)	367 (59.97%)	326 (60.48%)	97 (51.87%)
	17 to <18	178 (25.65%)	159 (25.98%)	139 (25.79%)	46 (24.60%)
	Missing	1 (0.14%)	1 (0.16%)	0 (0%)	0 (0%)
**Race/ethnicity**					
	White	321 (46.25%)	295 (48.20%)	259 (48.05%)	89 (47.59%)
	Black	19 (2.74%)	14 (2.29%)	11 (2.04%)	5 (2.67%)
	Latino/Hispanic	18 (2.59%)	15 (2.45%)	11 (2.04%)	3 (1.60%)
	Asian	89 (12.82%)	72 (11.77%)	65 (12.06%)	17 (9.09%)
	Multiracial	46 (6.63%)	43 (7.03%)	41 (7.61%)	19 (10.16%)
	Other	194 (27.95%)	168 (27.45%)	149 (27.64%)	54 (28.88%)
	Missing	7 (1.01%)	5 (0.82%)	3 (0.56%)	0 (0%)
**Sex**					
	Female	542 (78.10%)	478 (78.11%)	429 (79.59%)	150 (80.21%)
	Male	142 (20.46%)	125 (20.43%)	105 (19.48%)	36 (19.25%)
	Intersex	2 (0.29%)	1 (0.16%)	1 (0.19%)	0 (0%)
	Missing	9 (1.30%)	8 (1.31%)	4 (0.74%)	1 (0.53%)
**Gender identity differs from sex**					
	Yes	147 (21.18%)	124 (20.26%)	113 (20.97%)	45 (24.06%)
	No	489 (70.46%)	438 (71.57%)	385 (71.43%)	130 (69.52%)
	Unsure	48 (6.91%)	42 (6.86%)	37 (6.87%)	11 (5.89%)
	Missing	10 (1.44%)	8 (1.31%)	4 (0.74%)	1 (0.53%)
**Sexual orientation**					
	Heterosexual	295 (42.51%)	269 (43.95%)	235 (43.60%)	83 (44.39%)
	Homosexual	6 (0.86%)	6 (0.98%)	5 (0.93%)	0 (0%)
	Bisexual	104 (14.99%)	97 (15.85%)	85 (15.77%)	28 (14.97%)
	Queer	12 (1.73%)	12 (1.96%)	10 (1.86%)	4 (2.14%)
	Gay	12 (1.73%)	12 (1.96%)	10 (1.86%)	2 (1.07%)
	Lesbian	29 (4.18%)	26 (4.25%)	25 (4.64%)	8 (4.28%)
	Pansexual	32 (4.61%)	29 (4.74%)	28 (5.20%)	10 (5.35%)
	Asexual	19 (2.74%)	17 (2.78%)	15 (2.78%)	7 (3.74%)
	Other	31 (4.47%)	28 (4.58%)	24 (4.45%)	7 (3.74%)
	Unsure	55 (7.93%)	50 (8.17%)	47 (8.72%)	18 (9.63%)
	No response	50 (7.20%)	44 (7.19%)	38 (7.05%)	16 (8.56%)
	Missing	49 (7.06%)	22 (3.60%)	17 (3.15%)	4 (2.14%)

^a^SMFQ: Mood and Feelings Questionnaire—Short.

**Table 2:** Demographics and mean depressive symptoms among SSI completers, by selected SSI

Demographic characteristic				ABC Project, mean (SD) or N (%)	Project CARE, mean (SD) or N (%)		Project Personality, mean (SD) or N (%)	
N				75	38		74	
SMFQ^a^				17.47 (6.84)	16.24 (6.80)		15.69 (5.46)	
**Age**								
		<10	1 (1.33%)		2 (5.26%)	0		
		11 to 13	16 (21.33%)		9 (23.68%)	16 (21.62%)		
		14 to 16	47 (62.67%)		18 (47.37%)	32 (43.24%)		
		17 to <18	11 (14.67%)		9 (23.68%)	26 (35.14%)		
**Race/ethnicity**								
		White	33 (44.00%)		18 (47.37%)	38 (51.35%)		
		Black	3 (4.00%)		1 (2.63%)	1 (1.35%)		
		Latino/Hispanic	1 (1.33%)		0 (0%)	2 (2.70%)		
		Asian	4 (5.33%)		4 (10.53%)	9 (12.16%)		
		Other	26 (34.67%)		10 (26.32%)	18 (24.32%)		
		Multiple	8 (10.66%)		5 (13.15%)	6 (8.11%)		
**Sex**								
		Female	63 (85.14%)		28 (73.68%)	59 (79.73%)		
		Male	11 (14.86%)		10 (26.32%)	15 (20.27%)		
		Intersex	0 (0%)		0 (0%)	0 (0%)		
**Gender identity differs from sex**								
	Yes			20 (27.03%)	11 (28.95%)		14 (18.92%)	
	No			49 (66.22%)	25 (65.79%)		56 (75.68%)	
	Unsure			5 (6.76%)	2 (5.26%)		4 (5.41%)	
**Sexual orientation**								
	Heterosexual			27 (36.99%)	23 (62.16%)		33 (45.21%)	
	Homosexual			0 (0%)	0 (0%)		0 (0%)	
	Bisexual			10 (13.70%)	4 (10.81%)		14 (19.18%)	
	Queer			0 (0%)	3 (8.11%)		1 (1.37%)	
	Gay			1 (1.37%)	0 (0%)		1 (1.37%)	
	Lesbian			6 (8.22%)	1 (2.70%)		1 (1.37%)	
	Pansexual			7 (9.59%)	2 (5.41%)		1 (1.37%)	
	Asexual			3 (4.11%)	1 (2.70%)		3 (4.11%)	
	Other			4 (5.48%)	0 (0%)		3 (4.11%)	
	Unsure			8 (10.96%)	1 (2.70%)		9 (12.33%)	
	No response			7 (9.59%)	2 (5.41%)		7 (9.59%)	

^a^SMFQ: Mood and Feelings Questionnaire—Short.

On average, youths who completed YES in full spent 44.71 minutes on the YES website (SD 37.24 minutes; median 34.96 minutes; range 10.95-292.23 minutes), inclusive of all questionnaires, their chosen SSI, and writing anonymous coping advice for the YES Advice Center. Across all youths who accessed the website, including those who neither began nor completed an SSI, average time spent on YES was 27.92 minutes; this value did not differ significantly across the three programs.

#### Did Youths Perceive YES as Acceptable?

Youths who completed an SSI and the PFS (N=187), both collapsing across SSIs and within each SSI (Table 3; all item-level means >3.50/5), found YES acceptable. Overall, youths rated their chosen SSI as enjoyable (3.72/5.00), easy to understand (4.25/5.00), easy to use (4.31/5.00), likely to help their peers (4.04/5.00), and worth recommending to others (3.83/4.00). Youths also generally endorsed trying their hardest on the SSI (3.83/5.00) and agreement with the SSI’s message (4.29/5.00). Ratings on these items did not differ by SSI selection.

**Table 3:** Means and standard deviations of Program Feedback Scale items among SSI completers, by selected SSI and across all SSIs, mean (SD).

Item		ABC Project	Project CARE	Project Personality	All SSIs
Enjoy		3.57 (1.03)	3.84 (0.75)	3.81 (1.00)	3.72 (0.97)
Understood		4.16 (0.85)	4.08 (0.88)	4.43 (0.74)	4.25 (0.83)
Easy to use		4.28 (0.83)	4.21 (0.74)	4.39 (0.76)	4.31 (0.78)
Tried hardest		3.85 (1.09)	3.55 (1.08)	3.95 (1.03)	3.83 (1.07)
Helpful		3.97 (1.08)	4.16 (0.89)	4.05 (1.06)	4.04 (1.03)
Recommend to friend		3.72 (1.16)	3.95 (1.01)	3.89 (1.15)	3.83 (1.13)
Agree with message		4.11 (0.95)	4.47 (0.76)	4.38 (0.75)	4.29 (0.85)
Full scale		3.95 (0.82)	4.04 (0.61)	4.13 (0.69)	4.04 (0.73)

#### Did Hopelessness, Agency, Perceived Control, and Self-Hatred Improve From Before to After YES?

We tested SSI short-term utility both across SSIs, given their common structures and shared principles underlying each program’s development, and separately, as each SSI differed in content. Across SSIs, and for each SSI separately, effect sizes *d*_av_ and *d*_z_, 95% confidence intervals are reported (Table 4). Collapsing across SSIs, youths reported significant pre-to-post-SSI improvements in all proximal outcomes, regardless of the *d* computation approach. For overall reductions in self-hatred, small-to-medium effects emerged (*d*_av_=0.32, 95% CI 0.16, 0.47; *d*_z_=0.61, 95% CI 0.44, 0.77), with post-SSI self-hatred showing a 55% chance of being lower than pre-SSI self-hatred (per the “common language effect size” estimate; see [68]). For overall reductions in hopelessness, medium-to-large effects emerged (*d*_av_=0.53, 95% CI 0.37, 0.69; *d*_z_=0.71, 95% CI 0.54, 0.87), with post-SSI hopelessness showing a 62% chance of being lower than pre-SSI hopelessness. For overall improvements in agency, small-to-medium effects emerged (*d*_av_=0.39, 95% CI 0.24, 0.55; *d*_z_=0.50, 95% CI 0.34, 0.65), with post-SSI agency showing a 59% chance of being higher than the pre-SSI agency. For overall improvements in perceived control, medium-to-large effects emerged (*d*_av_=0.60, 95% CI 0.44, 0.76; *d*_z_=0.72, 95% CI 0.55, 0.89), with post-SSI self-hatred scores showing a 64% chance of being lower than pre-SSI scores. SSI-specific effect sizes fell within similar ranges (see Table 3). Within and across SSIs, 95% effect size confidence intervals overlapped across proximal outcomes, suggesting no detectable differences in youths’ pre-to-post-program changes in hopelessness, self-hatred, agency, or perceived control as a function of SSI selection.

**Table 4:** Means, standard deviations, and effect sizes by selected SSI; mean (SD) or (95% CI).

Outcome variable		ABC Project	Project CARE	Project Personality
**Agency**				
	Pre-SSI	4.36 (1.69)	4.66 (1.62)	4.69 (1.64)
	Post-SSI	5.03 (1.84)	5.42 (1.34)	5.24 (1.73)
	*d*_av_ (95% CI)	0.40 (0.15, 0.65)	0.58 (0.20, 0.95)	0.31 (0.07, 0.55)
	*d*_z_ (95% CI)	0.60 (0.34, 0.86)	0.65 (0.26, 1.02)	0.34 (0.10, 0.58)
**Hopelessness**				
	Pre-SSI	2.81 (0.82)	2.75 (0.79)	2.59 (0.86)
	Post-SSI	2.38 (0.89)	2.16 (0.80)	2.13 (0.76)
	*d*_av_	0.44 (0.19, 0.69)	0.83 (0.44, 1.21)	0.49 (0.25, 0.74)
	*d*_z_	0.62 (0.36, 0.87)	1.01 (0.60, 1.42)	0.66 (0.40, 0.91)
**Self-hate**				
	Pre-SSI	4.15 (1.74)	4.05 (1.70)	3.79 (1.74)
	Post-SSI	3.57 (1.65)	3.49 (1.80)	3.13 (1.80)
	*d*_av_	0.31 (0.06, 0.55)	0.35 (–0.02, 0.71)	0.31 (0.07, 0.55)
	*d*_z_	0.65 (0.38, 0.91)	0.72 (0.32, 1.11)	0.54 (0.29, 0.79)
**Perceived control**				
	Pre-SSI	6.72 (2.81)	6.43 (2.65)	6.32 (2.77)
	Post-SSI	5.12 (2.76)	4.69 (2.66)	4.90 (2.19)
	*d*_av_	0.54 (0.29, 0.79)	0.75 (0.35, 1.14)	0.59 (0.33, 0.85)
	*d*_z_	0.68 (0.41, 0.94)	1.03 (0.59, 1.45)	0.65 (0.38, 0.90)

#### Did Youths Subjectively Detect Changes in Hopelessness and Problem-Solving Ability From Before to After YES?

Collapsing across SSIs, at post-intervention, 15.6% (N=29) of youths reported feeling “much less hopeless” compared to before beginning the SSI; 53.1% (N=99) felt “a little less hopeless”; 27.9% (N=52) felt “the same amount hopeless”; 3.4% (N=6) felt “a little more hopeless”; and 0.0% (N=0) felt “a lot more hopeless.” Separately, 8.4% (N=16) of youths reported feeling “much more able to solve problems” compared to before beginning the SSI; 50.0% (N=93) felt “a little more able to solve problems”; 38.8% (N=72) felt “the same amount able to solve problems”; 2.8% (N=5) felt “a little less able to solve problems”; and 0.0% (N=0) felt “a lot less able to solve problems.”

## Discussion

This nonexperimental study evaluated the perceived acceptability and short-term effects of Project YES, an open-access platform for youths offering three 30-minute, self-directed SSIs, each teaching a strategy for coping with internalizing distress. Regardless of SSI selection, youths who completed an SSI reported significant pre-to-post-program reductions in hopelessness (*d*_av_=0.53; *d*_z_=0.71), self-hate (*d*_av_=0.32; *d*_z_=0.61), perceived control (*d*_av_=0.60; *d*_z_=0.72) and agency (*d*_av_=0.39; *d*_z_=0.50). Youths who completed an SSI rated it as enjoyable, easy to understand, likely to help peers, and worth recommending to others, and there was no evidence for adverse SSI effects (eg, no youths reported feeling “much more hopeless” or “much less able to solve problems” after program completion). Based on largely overlapping confidence intervals for *d*_av_ and *d*_z_, no evidence emerged for differential SSI effects on proximal outcomes.

Findings reinforce and extend evidence supporting the utility of SSIs for youth experiencing internalizing distress [19,20,53]. Past studies have raised the possibility that open-access, online SSIs might benefit youths experiencing elevated symptoms. However, no studies have tested their acceptability or utility in naturalistic settings (versus in controlled trials, which offer participation incentives, necessitate parental involvement, and prevent youth from choosing which SSIs they wish to receive). This study provides the first evidence that online SSIs may be acceptable—and potentially helpful—when delivered in the “real-world” as free, publicly available supports. Within-group post-SSI effects were modest; even so, the accessibility, cost-free nature, and brevity of YES suggest its public health value, particularly if available continuously and on a large scale. The self-selected youth sample reported elevated depressive symptoms (among all youths who started YES, SMFQ mean 17.06, SD 6.40, and among youths who completed a YES SSI, SMFQ mean 16.51, SD 6.34, both >50% higher than commonly used screening cut-offs for adolescent depression [69]), indicating the acceptability and possible utility of YES among youths with immediate clinical needs.

In addition to frequently reporting clinically elevated depressive symptoms, many youths accessing YES reported one or more marginalized identities, including sexual minority status (50.13% of users) and racial/ethnic minority status (53.28% of users). YES includes no exclusion or screening criteria—youths from these communities *self-selected* into participation—and no formal efforts were made to recruit members of specific groups. Thus, YES and similar platforms may represent efficient avenues for providing support to youths that may be likely to need, but unlikely to access traditional services. Simultaneously, more female than male adolescents self-selected into YES. This discrepancy may reflect higher rates of depression among adolescent girls versus boys, but it also fits with research suggesting that boys who are experiencing depression access treatment less often than girls [70]. Future work may focus on redesigning YES-like platforms that appeal to adolescents regardless of sex.

Regarding usability, more than 34% of youths who began an SSI finished it. This rate compares favorably with prior studies of “real-world” engagement with self-directed, tech-based psychological supports (1-28% retention) [15,70], and even with rates of retention in outpatient youth psychotherapy (premature drop-out rates range from 16-75%) [71,72]. However, Project YES is the first naturalistic evaluation of web-based, youth-directed SSIs; thus, further research is needed to gauge whether a 34% completion rate is typical of this type of program, as well as the degree to which this rate could be improved. Systematically testing strategies for further improving retention in YES-like platforms will be valuable—especially among older users (above age 14), for whom completion rates were lower than for younger users (ages 13 and younger). Retention may be improved by eliminating or minimizing questionnaires (all YES questionnaires are optional, but their presence may be a deterrence) and decreasing intervention length and iteratively re-testing ‘hyper-brief’ SSIs for acceptability and utility. Other revisions may include personalizing intervention content for youths of different age ranges or developmental stages and optimizing YES for smartphone-based completion (currently, YES is completable across internet-equipped devices but is optimized for desktop and laptop users).

No evidence emerged for differential SSI effects on hopelessness, perceived control, agency, or self-hatred, although each SSI was initially intended to target a different proximal outcome. Project CARE was designed to reduce self-hatred, Project Personality was designed to strengthen perceived control and combat hopelessness, and the ABC Project was intended to strengthen perceived agency over behavior. However, no evidence emerged for domain-specific effects across SSIs. This outcome, while quite preliminary, fits with existing evidence that change-mechanisms may be more similar than different across psychotherapies [19,73]. Here, each SSI was designed according to a common set of principles [19] and included several elements in common (eg, neuroscience-based psychoeducation; testimonials from scientists and peers; opportunities to offer advice to others), which themselves may promote a shared set of adaptive thinking styles.

A second possibility is that the constructs assessed in this study—and clinical psychology intervention research more broadly—have shown considerable overlap, both conceptually and statistically [74,75], and may not reflect entirely distinct constructs. Additional measurement-focused work is needed to determine the extent to which self-report assessments of agency, perceived control, hopelessness, self-hate, and numerous other “thinking styles” commonly studied in intervention research reflect genuinely distinct factors, versus features of a shared latent construct.

A third possibility involves the role of *personal choice* on the platform. YES users independently decide not only whether to participate, but which SSI to complete and whether to publicly share anonymous advice with peers. These opportunities to exert agency, common across YES SSIs, may contribute to similar cross-program outcomes, at least proximally. This possibility is especially relevant to adolescents, for whom autonomy and assertion are both highly motivating and key developmental tasks. Future work may formally test how personal choice and opportunities to develop autonomy shape adolescents’ receptivity and response to YES-like support—and to mental health treatment more broadly.

Study limitations suggest directions for future research. First, this study is nonexperimental, observational, and anonymous. All of these features are necessary given the study’s objectives (to evaluate acceptability and utility of SSIs in naturalistic settings), but results should be interpreted with caution. For instance, despite targeted social media-based advertising, it is impossible to determine with certainty that participants were sincere when reporting their age-ranges. Further, as in any non-randomized program evaluation, possible selection bias cannot be overlooked. For instance, youths who self-initiate and complete a given SSI may be those most likely to benefit from it; thus, present findings may not reflect the SSIs’ utility for *all* youths. Future randomized trials that proactively address selection bias are needed to unpack this possibility. Third, given the anonymous nature of YES, we could not follow-up with participants; however, prior trials suggest these and similar SSIs confer longer-term clinical benefits [19,42,53]. Additionally, our SSIs are available only in English at this time, limiting access for large portions of the global population. Translation and further pilot-testing are needed to begin gauging the cross-contextual, cross-cultural promise of YES and similar platforms.

Overall, results suggest that open-access, free of charge SSIs for adolescents ages 11-17 are acceptable; associated with proximal improvements in hopelessness, agency, perceived control, and self-hatred; and show no evidence for adverse effects. Overall, YES users were more likely to perceive *some* psychological benefits than *none*—either in hopelessness, problem-solving skills, or both. Certainly, YES-like platforms cannot and should not replace longer-term psychotherapies—but they may offer much-needed low-cost, easily accessible support to the many youths who might otherwise go without treatment. Future work may examine the broader-scale use of YES across cultures, integration of YES-like platforms with more intensive forms of support, and systematically examine the role of SSI choice in adolescents’ response to similar interventions.

## Appendix

Multimedia Appendix 1 Project YES Analytic Code and Supplementary Analyses.

## Abbreviations

PFS: Program Feedback Scale
SMFQ: Mood and Feelings Questionnaire—Short
SSI: single session intervention
YES: Youth Empowerment & Support

## References

[1] Kazdin AE, Rabbitt SM (2013). Novel Models for Delivering Mental Health Services and Reducing the Burdens of Mental Illness. Clinical Psychological Science.

[2] Konrad TR, Ellis AR, Thomas KC, Holzer CE, Morrissey JP (2009). County-Level Estimates of Need for Mental Health Professionals in the United States. PS.

[3] Powell AC, Torous JB, Firth J, Kaufman KR (2020). Generating value with mental health apps. BJPsych Open.

[4] Torous J, Wisniewski H, Liu G, Keshavan M (2018). Mental Health Mobile Phone App Usage, Concerns, and Benefits Among Psychiatric Outpatients: Comparative Survey Study. JMIR Ment Health.

[5] Pew RC Teens, Social Media & Technology 2018 Internet. Pew Research Center.

[6] Firth J, Torous J, Nicholas J, Carney R, Pratap A, Rosenbaum S, Sarris J (2017). The efficacy of smartphone-based mental health interventions for depressive symptoms: a meta-analysis of randomized controlled trials. World Psychiatry.

[7] Kerst A, Zielasek J, Gaebel W (2020). Smartphone applications for depression: a systematic literature review and a survey of health care professionals' attitudes towards their use in clinical practice. Eur Arch Psychiatry Clin Neurosci.

[8] Christie GI, Shepherd M, Merry SN, Hopkins S, Knightly S, Stasiak K (2019). Gamifying CBT to deliver emotional health treatment to young people on smartphones. Internet Interv.

[9] Bevan Jones R, Thapar A, Rice F, Mars B, Agha SS, Smith D, Merry S, Stallard P, Thapar AK, Jones I, Simpson SA (2020). A Digital Intervention for Adolescent Depression (MoodHwb): Mixed-Methods Feasibility Evaluation. JMIR Ment Health.

[10] Cliffe B, Croker A, Denne M, Smith J, Stallard P (2020). Digital Cognitive Behavioral Therapy for Insomnia for Adolescents With Mental Health Problems: Feasibility Open Trial. JMIR Ment Health.

[11] Fleming TM, de Beurs D, Khazaal Y, Gaggioli A, Riva G, Botella C, Baños RM, Aschieri F, Bavin LM, Kleiboer A, Merry S, Lau HM, Riper H (2016). Maximizing the Impact of e-Therapy and Serious Gaming: Time for a Paradigm Shift. Front Psychiatry.

[12] Fleming TM, Bavin L, Stasiak K, Hermansson-Webb E, Merry SN, Cheek C, Lucassen M, Lau HM, Pollmuller B, Hetrick S (2016). Serious Games and Gamification for Mental Health: Current Status and Promising Directions. Front Psychiatry.

[13] Wasil AR, Venturo-Conerly KE, Shingleton RM, Weisz JR (2019). A review of popular smartphone apps for depression and anxiety: Assessing the inclusion of evidence-based content. Behav Res Ther.

[14] Punukollu M, Marques M (2019). Use of mobile apps and technologies in child and adolescent mental health: a systematic review. Evid Based Ment Health.

[15] Fleming T, Bavin L, Lucassen M, Stasiak K, Hopkins S, Merry S (2018). Beyond the Trial: Systematic Review of Real-World Uptake and Engagement With Digital Self-Help Interventions for Depression, Low Mood, or Anxiety. J Med Internet Res.

[16] Liverpool S, Mota CP, Sales CMD, Čuš A, Carletto S, Hancheva C, Sousa S, Cerón SC, Peral PM, Pietrabissa G, Moltrecht B, Ulberg R, Ferreira N, Edbrooke-Childs J (2020). Engaging Children and Young People in Digital Mental Health Interventions: Systematic Review of Modes of Delivery, Facilitators, and Barriers. J Med Internet Res.

[17] Schleider JL, Weisz JR (2017). Little Treatments, Promising Effects? Meta-Analysis of Single-Session Interventions for Youth Psychiatric Problems. J Am Acad Child Adolesc Psychiatry.

[18] Weisz JR, Kuppens S, Ng MY, Eckshtain D, Ugueto AM, Vaughn-Coaxum R, Jensen-Doss A, Hawley KM, Krumholz Marchette LS, Chu BC, Weersing VR, Fordwood SR (2017). What five decades of research tells us about the effects of youth psychological therapy: A multilevel meta-analysis and implications for science and practice. Am Psychol.

[19] Schleider JL, Dobias ML, Sung JY, Mullarkey MC (2020). Future Directions in Single-Session Youth Mental Health Interventions. J Clin Child Adolesc Psychol.

[20] Schleider J, Weisz J (2018). A single-session growth mindset intervention for adolescent anxiety and depression: 9-month outcomes of a randomized trial. J Child Psychol Psychiatry.

[21] Schleider JL, Burnette JL, Widman L, Hoyt C, Prinstein MJ (2019). Randomized Trial of a Single-Session Growth Mind-Set Intervention for Rural Adolescents' Internalizing and Externalizing Problems. J Clin Child Adolesc Psychol.

[22] Miu AS, Yeager DS (2014). Preventing Symptoms of Depression by Teaching Adolescents That People Can Change. Clinical Psychological Science.

[23] Project YES: Youth Empowerment & Support. Lab for Scalable Mental Health.

[24] Pössel P, Smith E (2020). Integrating Beck's Cognitive Theory of Depression and the Hopelessness Model in an Adolescent Sample. J Abnorm Child Psychol.

[25] Gibb BE, Alloy LB (2006). A prospective test of the hopelessness theory of depression in children. J Clin Child Adolesc Psychol.

[26] Rubenstein LM, Alloy LB, Abramson LY, Reich JW, Infurna FJ (2016). Perceived Control and Depression: Forty Years of Research. Perceived Control: Theory, Research, and Practice in the First 50 Years.

[27] Moè A (2015). Perceived Control Mediates the Relations between Depressive Symptoms and Academic Achievement in Adolescence. Span J Psychol.

[28] Jose PE, Weir KF (2013). Adolescent sense of control: a downward extension of the Shapiro Control Inventory to pre- and early adolescents. J Genet Psychol.

[29] Schleider JL, Abel MR, Weisz JR (2019). Do Immediate Gains Predict Long-Term Symptom Change? Findings from a Randomized Trial of a Single-Session Intervention for Youth Anxiety and Depression. Child Psychiatry Hum Dev.

[30] Dobias ML, Mullarkey MC, Schleider JL (2019). The Teenage Goals Project. Open Science Framework.

[31] Turnell AI, Fassnacht DB, Batterham PJ, Calear AL, Kyrios M (2019). The Self-Hate Scale: Development and validation of a brief measure and its relationship to suicidal ideation. J Affect Disord.

[32] Fox KR, Ribeiro JD, Kleiman EM, Hooley JM, Nock MK, Franklin JC (2018). Affect toward the self and self-injury stimuli as potential risk factors for nonsuicidal self-injury. Psychiatry Res.

[33] Pfeiffer PN, Brandfon S, Garcia E, Duffy S, Ganoczy D, Myra Kim H, Valenstein M (2014). Predictors of suicidal ideation among depressed Veterans and the interpersonal theory of suicide. J Affect Disord.

[34] Zelkowitz RL, Cole DA (2019). Self-Criticism as a Transdiagnostic Process in Nonsuicidal Self-Injury and Disordered Eating: Systematic Review and Meta-Analysis. Suicide Life Threat Behav.

[35] Smith DM, Wang SB, Carter ML, Fox KR, Hooley JM (2020). Longitudinal predictors of self-injurious thoughts and behaviors in sexual and gender minority adolescents. J Abnorm Psychol.

[36] Halamová J, Kanovský M, Jurková V, Kupeli N (2018). Effect of a Short-Term Online Version of a Mindfulness-Based Intervention on Self-criticism and Self-compassion in a Nonclinical Sample. SP.

[37] Luoma JB, Platt MG (2015). Shame, self-criticism, self-stigma, and compassion in Acceptance and Commitment Therapy. Current Opinion in Psychology.

[38] Hooley JM, Fox KR, Wang SB, Kwashie AND (2018). Novel online daily diary interventions for nonsuicidal self-injury: a randomized controlled trial. BMC Psychiatry.

[39] Duarte C, Gilbert P, Stalker C, Catarino F, Basran J, Scott S, Horgan G, Stubbs RJ (2019). Effect of adding a compassion-focused intervention on emotion, eating and weight outcomes in a commercial weight management programme. J Health Psychol.

[40] Schleider J, Mullarkey M, Mumper E, Sung J (2019). The ABC Project. Open Science Framework.

[41] Snyder C, Sympson S, Ybasco F, Borders T, Babyak M, Higgins R (2011). State Hope Scale. PsycTESTS Dataset.

[42] Gawrysiak M, Nicholas C, Hopko DR (2009). Behavioral activation for moderately depressed university students: Randomized controlled trial. Journal of Counseling Psychology.

[43] Ritschel LA, Ramirez CL, Jones M, Craighead WE (2011). Behavioral Activation for Depressed Teens: A Pilot Study. Cognitive and Behavioral Practice.

[44] Pass L, Lejuez CW, Reynolds S (2018). Brief Behavioural Activation (Brief BA) for Adolescent Depression: A Pilot Study. Behav Cogn Psychother.

[45] Chu BC, Colognori D, Weissman AS, Bannon K (2009). An Initial Description and Pilot of Group Behavioral Activation Therapy for Anxious and Depressed Youth. Cognitive and Behavioral Practice.

[46] Duckworth AL (2019). Using Psychological Science to Help Children Thrive. Perspect Psychol Sci.

[47] Eskreis-Winkler L, Milkman KL, Gromet DM, Duckworth AL (2019). A large-scale field experiment shows giving advice improves academic outcomes for the advisor. Proc Natl Acad Sci U S A.

[48] Eskreis-Winkler L, Fishbach A, Duckworth AL (2018). Dear Abby: Should I Give Advice or Receive It?. Psychol Sci.

[49] Resnick B (2019). These scientists want to make psychotherapy sessions much, much shorter Internet. Vox.

[50] YES (Youth Empowerment & Support). Open Science Framework.

[51] Brahmbhatt K, Grupp-Phelan J (2019). Parent-Adolescent Agreement About Adolescent's Suicidal Thoughts: A Divergence. Pediatrics.

[52] Orchard F, Pass L, Cocks L, Chessell C, Reynolds S (2019). Examining parent and child agreement in the diagnosis of adolescent depression. Child Adolesc Ment Health.

[53] Schleider J, Mullarkey M, Chacko A (2020). Harnessing Wise Interventions to Advance the Potency and Reach of Youth Mental Health Services. Clin Child Fam Psychol Rev.

[54] Aronson E (1999). The power of self-persuasion. American Psychologist.

[55] Schleider JL, Weisz JR (2019). Project Personality. Open Science Framework.

[56] Schleider JL, Weisz JR (2016). Reducing risk for anxiety and depression in adolescents: Effects of a single-session intervention teaching that personality can change. Behav Res Ther.

[57] Lundervold AJ, Hinshaw SP, Sørensen Lin, Posserud M (2016). Co-occurring symptoms of attention deficit hyperactivity disorder (ADHD) in a population-based sample of adolescents screened for depression. BMC Psychiatry.

[58] Snyder CR, Sympson SC, Ybasco FC, Borders TF, Babyak MA, Higgins RL (1996). Development and validation of the State Hope Scale. J Pers Soc Psychol.

[59] Perczel Forintos D, Rózsa S, Pilling J, Kopp M (2013). Proposal for a short version of the Beck Hopelessness Scale based on a national representative survey in Hungary. Community Ment Health J.

[60] Rhoades H, Rusow JA, Bond D, Lanteigne A, Fulginiti A, Goldbach JT (2018). Homelessness, Mental Health and Suicidality Among LGBTQ Youth Accessing Crisis Services. Child Psychiatry Hum Dev.

[61] Turnell AI, Fassnacht DB, Batterham PJ, Calear AL, Kyrios M (2019). The Self-Hate Scale: Development and validation of a brief measure and its relationship to suicidal ideation. J Affect Disord.

[62] Anvari F, Lakens D (2019). Using Anchor-Based Methods to Determine the Smallest Effect Size of Interest. PsyArXiv Preprints.

[63] Schleider JL, Mullarkey MC, Weisz JR (2019). Virtual Reality and Web-Based Growth Mindset Interventions for Adolescent Depression: Protocol for a Three-Arm Randomized Trial. JMIR Res Protoc.

[64] Schleider J, Dobias M, Fassler J, Shroff A, Pati S (2020). Promoting Treatment Access Following Pediatric Primary Care Depression Screening: Randomized Trial of Web-Based, Single-Session Interventions for Parents and Youths. Journal of the American Academy of Child & Adolescent Psychiatry.

[65] Dobias M, Chen S, Fox K, Schleider J (2020). Brief interventions for self-injurious thoughts and behaviors in young people: A systematic review. Open Science Framework.

[66] Mullarkey M, Dobias M, Sung J, Shumake J, Beevers C, Schleider J (2020). A scalable, single session intervention for perceived control over anxiety during COVID-19. Open Science Framework.

[67] Schleider Jessica, Sung Jenna, Bianco Amanda, Gonzalez Adam, Vivian Dina, Mullarkey Michael (2020). Open Pilot Trial of a Single-Session Consultation Service for Clients on Psychotherapy Wait-Lists. The Behavior Therapist.

[68] Lakens D (2013). Calculating and reporting effect sizes to facilitate cumulative science: a practical primer for t-tests and ANOVAs. Front Psychol.

[69] Stoep AV, McCauley E, Thompson KA, Herting JR, Kuo ES, Stewart DG, Anderson CA, Kushner S (2005). Universal Emotional Health Screening at the Middle School Transition. J Emot Behav Disord.

[70] Lu W (2019). Adolescent Depression: National Trends, Risk Factors, and Healthcare Disparities. Am J Health Behav.

[71] Baruch G, Vrouva I, Fearon P (2009). A Follow-up Study of Characteristics of Young People that Dropout and Continue Psychotherapy: Service Implications for a Clinic in the Community. Child and Adolescent Mental Health.

[72] de Haan AM, Boon AE, de Jong JTVM, Hoeve M, Vermeiren RRJM (2013). A meta-analytic review on treatment dropout in child and adolescent outpatient mental health care. Clin Psychol Rev.

[73] Cuijpers P, Reijnders M, Huibers MJH (2019). The Role of Common Factors in Psychotherapy Outcomes. Annu Rev Clin Psychol.

[74] Mullarkey MC, Schleider JL (2020). Contributions of fixed mindsets and hopelessness to anxiety and depressive symptoms: A commonality analysis approach. J Affect Disord.

[75] Naragon-Gainey K, McMahon TP, Park J (2018). The contributions of affective traits and emotion regulation to internalizing disorders: Current state of the literature and measurement challenges. Am Psychol.

