# Supplementary material for: Acceptability and Utility of an Open-Access, Online Single-Session Intervention Platform for Adolescent Mental Health
Source: JMIR Ment Health. 2020 Jun 30;7(6):e20513. doi: 10.2196/20513 (PMC7367540; doi:10.2196/20513)
Supplement: Multimedia Appendix 1 [file mental_v7i6e20513_app1.docx]

**Online Supplement: Logistic Regressions and Elastic Nets**

## **Pre-Registered Logistic Regressions**

## **Predicting Completing 85% or More of the Survey**

The logistic regression can’t be interpreted due to difficulties with collinearity, specifically that biological sex and gender identity are so co-linear we can’t reliably interpret any of the results. This is true for all pre-registered logistic regressions. We therefore deviate from our pre-registration and analyze these predictions using an elastic net in each case.

An elastic net is a penalized version of the linear model that combines lasso (dropping predictors) and ridge (shrinking predictor coefficients toward one another) penalties to generate the best possible predictions of the outcome variable “out of sample.” We can estimate the model’s performance out of sample by using 10-fold cross-validation. 10-fold cross-validation involves splitting the data up into 10 sections, estimating the elastic net in 9 of those sections, and seeing how well the elastic net estimated on those 9 sections predicts the outcome in the 10th section the elastic net model hasn’t “seen.” This process is then repeated 10 times to minimize the potential bias of using just one “out of sample” section of the data. This can also lead to predictions of negative r-squared variance, as at least some models may be a worse prediction than just predicting the mean value of the outcome for every single observation (or the equivalent of drawing a horizotnal regression line through the data). We also use nested cross-validation to determine the optimal mixture of lasso (dropping predictors) and ridge (shrinking predictor coefficients toward one another) penalties in the elastic net, as otherwise the predictions can be optimistically biased.

See <doi:10.1111/j.1467-9868.2005.00503.x> for more information on the elastic net and <doi:10.1001/jamapsychiatry.2019.3671> for establishment of best practices for prediction including the use of 10-fold cross-validation rather than other forms of cross-validation.

Using this procedure, we only predict 0.1% of the variance in who completes the survey with our pre-registered predictors (age range, biological sex, gender identity, and baseline MFQ depression sum score).

## Making sure all the factor levels and descriptives of the predictors make sense

# levels(yes_data_pre_post_recoded_youth_only$age_range_factor)
# levels(yes_data_pre_post_recoded_youth_only$bio_sex_factor)
# levels(yes_data_pre_post_recoded_youth_only$gender_identity_factor)
# psych::describe(yes_data_pre_post_recoded_youth_only$b_mfq_mean)

## Running the logistic regression

yes_data_pre_post_recoded_youth_only_survey_completion <- yes_data_pre_post_recoded_youth_only %>%
 dplyr::select(survey_85_percent_complete, age_range_factor, bio_sex_factor, gender_identity_factor, b_mfq_mean) %>% na.omit()

predict_survey_completion_glm <- glm(survey_85_percent_complete ~ ., data = yes_data_pre_post_recoded_youth_only_survey_completion, family = "binomial")

## Way too colinear to run these in a traditional logistic regression

check_collinearity(predict_survey_completion_glm)

## # Check for Multicollinearity

## Have to deviate and use a method that's more robust to collinearity

## Let's switch to an elastic net with a binomial outcome/nested cross-validation
tic()
predict_survey_completion_el_net <- beset_elnet(survey_85_percent_complete ~ ., data = yes_data_pre_post_recoded_youth_only_survey_completion, family = "binomial", seed = 33, nest_cv = T)
predict_survey_completion_el_net

##
## Results of nested 10-fold cross-validation repeated 10 times
## =======================================================
## Most conservative tuning parameters within
## 1 SE of best cross-validation Mean Cross Entropy:
## Mean S.E. Min Max
## alpha 0.966 0.046 0.892 0.990
## lambda 0.147 0.207 0.052 0.538
##
##
## Non-zero coefficients ranked in order of importance:
## Stnd.Coef.
## age_range_factor14 to 16 -0.04710
## gender_identity_factorMale,Female to male transgender/FTM 0.00070
## age_range_factor10 or younger 0.00050
## gender_identity_factorFemale,Not sure 0.00040
## gender_identity_factorFemale,Androgynous 0.00040
## gender_identity_factorNot sure -0.00020
## gender_identity_factorGenderqueer -0.00010
## gender_identity_factorMale,Female to male transgender/FTM,Trans male/Trans masculine 0.00010
## gender_identity_factorMale,Female,Genderqueer,Androgynous,Nonbinary,Agender 0.00010
## gender_identity_factorMale,Female,Transgender 0.00010
## gender_identity_factorMale,Transgender,Female to male transgender/FTM 0.00010
## S.E.
## age_range_factor14 to 16 0.01341
## gender_identity_factorMale,Female to male transgender/FTM 0.00123
## age_range_factor10 or younger 0.00122
## gender_identity_factorFemale,Not sure 0.00093
## gender_identity_factorFemale,Androgynous 0.00088
## gender_identity_factorNot sure 0.00056
## gender_identity_factorGenderqueer 0.00034
## gender_identity_factorMale,Female to male transgender/FTM,Trans male/Trans masculine 0.00018
## gender_identity_factorMale,Female,Genderqueer,Androgynous,Nonbinary,Agender 0.00018
## gender_identity_factorMale,Female,Transgender 0.00018
## gender_identity_factorMale,Transgender,Female to male transgender/FTM 0.00018
## Min
## age_range_factor14 to 16 -0.06110
## gender_identity_factorMale,Female to male transgender/FTM 0.00000
## age_range_factor10 or younger 0.00000
## gender_identity_factorFemale,Not sure 0.00000
## gender_identity_factorFemale,Androgynous 0.00000
## gender_identity_factorNot sure -0.00180
## gender_identity_factorGenderqueer -0.00110
## gender_identity_factorMale,Female to male transgender/FTM,Trans male/Trans masculine 0.00000
## gender_identity_factorMale,Female,Genderqueer,Androgynous,Nonbinary,Agender 0.00000
## gender_identity_factorMale,Female,Transgender 0.00000
## gender_identity_factorMale,Transgender,Female to male transgender/FTM 0.00000
## Max
## age_range_factor14 to 16 -0.02539
## gender_identity_factorMale,Female to male transgender/FTM 0.00498
## age_range_factor10 or younger 0.00388
## gender_identity_factorFemale,Not sure 0.00384
## gender_identity_factorFemale,Androgynous 0.00272
## gender_identity_factorNot sure 0.00000
## gender_identity_factorGenderqueer 0.00000
## gender_identity_factorMale,Female to male transgender/FTM,Trans male/Trans masculine 0.00056
## gender_identity_factorMale,Female,Genderqueer,Androgynous,Nonbinary,Agender 0.00056
## gender_identity_factorMale,Female,Transgender 0.00056
## gender_identity_factorMale,Transgender,Female to male transgender/FTM 0.00056
##
##
## Prediction Metrics:
## Deviance Explained S.E. Min Max
## Train Sample 0.003 0.001 0.002 0.005
## CV-Tune Holdout 0.001 0.001 0.000 0.002
## CV-Test Holdout 0.001 0.002 -0.001 0.002
## =======================================================

beep(sound = 3)
toc()

## 315.742 sec elapsed

### **Predicting Who Chose Which SSI**

Using elastic nets, we predict 0.5% of the variance in who selects Project Personality, 0.3% in who selects Project ABC, and -0.2% in who selects Project CARE using pre-registered predictors (age range, biological sex, gender identity, and baseline MFQ depression sum score).

## For Project Personality

yes_data_pre_post_recoded_youth_only_project_personality_selection <- yes_data_pre_post_recoded_youth_only %>%
 dplyr::select(ssi_choice_factor, age_range_factor, bio_sex_factor, gender_identity_factor, b_mfq_mean) %>% na.omit() %>%
 mutate(ssi_choice_character = as.character(ssi_choice_factor),
 project_personality_choice_character = case_when(
 ssi_choice_character == "Project Personality" ~ "Yes",
 ssi_choice_character == "BA-SSI" ~ "No",
 ssi_choice_character == "Self-Hate SSI" ~ "No"),
 project_personality_choice_factor = as.factor(project_personality_choice_character)) %>%
 dplyr::select(-ssi_choice_factor,-ssi_choice_character,-project_personality_choice_character)

predict_project_personality_glm <- glm(project_personality_choice_factor ~ ., data = yes_data_pre_post_recoded_youth_only_project_personality_selection, family = "binomial")

## Way too colinear to run these in a traditional logistic regression

check_collinearity(predict_project_personality_glm)

## # Check for Multicollinearity

## Have to deviate and use a method that's more robust to collinearity

## Let's switch to an elastic net with a binomial outcome/nested cross-validation
tic()
predict_project_personality_choice_el_net <- beset_elnet(project_personality_choice_factor ~ ., data = yes_data_pre_post_recoded_youth_only_project_personality_selection, family = "binomial", seed = 33, nest_cv = T)
predict_project_personality_choice_el_net

##
## Results of nested 10-fold cross-validation repeated 10 times
## =======================================================
## Most conservative tuning parameters within
## 1 SE of best cross-validation Mean Cross Entropy:
## Mean S.E. Min Max
## alpha 0.985 0.015 0.941 0.990
## lambda 0.035 0.003 0.032 0.042
##
##
## Non-zero coefficients ranked in order of importance:
## Stnd.Coef.
## gender_identity_factorNot sure 0.07150
## b_mfq_mean -0.05520
## age_range_factor10 or younger -0.02220
## gender_identity_factorMale,Female to male transgender/FTM 0.01040
## gender_identity_factorGenderqueer -0.00530
## gender_identity_factorFemale,Not sure -0.00070
## gender_identity_factorFemale,Transgender 0.00060
## gender_identity_factorFemale,Androgynous,Two-spirited 0.00050
## gender_identity_factorMale,Not sure 0.00050
## gender_identity_factorMale,Transgender,Female to male transgender/FTM,Trans male/Trans masculine,Genderqueer 0.00050
## gender_identity_factorOther (Please specify) 0.00050
## gender_identity_factorMale,Transgender,Female to male transgender/FTM,Trans male/Trans masculine -0.00050
## gender_identity_factorGenderqueer,Nonbinary 0.00040
## gender_identity_factorFemale,Genderqueer,Not sure 0.00040
## gender_identity_factorMale,Female to male transgender/FTM,Trans male/Trans masculine 0.00040
## gender_identity_factorGenderqueer,Gender expansive,Agender,Other (Please specify) 0.00030
## gender_identity_factorFemale 0.00020
## gender_identity_factorTransgender,Genderqueer -0.00010
## gender_identity_factorFemale,Androgynous -0.00010
## gender_identity_factorMale to female transgender/MTF,Trans female/Trans feminine -0.00010
## gender_identity_factorNonbinary 0.00010
## S.E.
## gender_identity_factorNot sure 0.00840
## b_mfq_mean 0.00912
## age_range_factor10 or younger 0.00580
## gender_identity_factorMale,Female to male transgender/FTM 0.00371
## gender_identity_factorGenderqueer 0.00247
## gender_identity_factorFemale,Not sure 0.00092
## gender_identity_factorFemale,Transgender 0.00093
## gender_identity_factorFemale,Androgynous,Two-spirited 0.00083
## gender_identity_factorMale,Not sure 0.00079
## gender_identity_factorMale,Transgender,Female to male transgender/FTM,Trans male/Trans masculine,Genderqueer 0.00079
## gender_identity_factorOther (Please specify) 0.00074
## gender_identity_factorMale,Transgender,Female to male transgender/FTM,Trans male/Trans masculine 0.00084
## gender_identity_factorGenderqueer,Nonbinary 0.00065
## gender_identity_factorFemale,Genderqueer,Not sure 0.00067
## gender_identity_factorMale,Female to male transgender/FTM,Trans male/Trans masculine 0.00070
## gender_identity_factorGenderqueer,Gender expansive,Agender,Other (Please specify) 0.00064
## gender_identity_factorFemale 0.00073
## gender_identity_factorTransgender,Genderqueer 0.00032
## gender_identity_factorFemale,Androgynous 0.00038
## gender_identity_factorMale to female transgender/MTF,Trans female/Trans feminine 0.00024
## gender_identity_factorNonbinary 0.00022
## Min
## gender_identity_factorNot sure 0.05830
## b_mfq_mean -0.06350
## age_range_factor10 or younger -0.02820
## gender_identity_factorMale,Female to male transgender/FTM 0.00380
## gender_identity_factorGenderqueer -0.00900
## gender_identity_factorFemale,Not sure -0.00250
## gender_identity_factorFemale,Transgender 0.00000
## gender_identity_factorFemale,Androgynous,Two-spirited 0.00000
## gender_identity_factorMale,Not sure 0.00000
## gender_identity_factorMale,Transgender,Female to male transgender/FTM,Trans male/Trans masculine,Genderqueer 0.00000
## gender_identity_factorOther (Please specify) 0.00000
## gender_identity_factorMale,Transgender,Female to male transgender/FTM,Trans male/Trans masculine -0.00210
## gender_identity_factorGenderqueer,Nonbinary 0.00000
## gender_identity_factorFemale,Genderqueer,Not sure 0.00000
## gender_identity_factorMale,Female to male transgender/FTM,Trans male/Trans masculine 0.00000
## gender_identity_factorGenderqueer,Gender expansive,Agender,Other (Please specify) 0.00000
## gender_identity_factorFemale 0.00000
## gender_identity_factorTransgender,Genderqueer -0.00070
## gender_identity_factorFemale,Androgynous -0.00120
## gender_identity_factorMale to female transgender/MTF,Trans female/Trans feminine -0.00060
## gender_identity_factorNonbinary 0.00000
## Max
## gender_identity_factorNot sure 0.08130
## b_mfq_mean -0.03820
## age_range_factor10 or younger -0.01270
## gender_identity_factorMale,Female to male transgender/FTM 0.01420
## gender_identity_factorGenderqueer -0.00150
## gender_identity_factorFemale,Not sure 0.00000
## gender_identity_factorFemale,Transgender 0.00200
## gender_identity_factorFemale,Androgynous,Two-spirited 0.00180
## gender_identity_factorMale,Not sure 0.00170
## gender_identity_factorMale,Transgender,Female to male transgender/FTM,Trans male/Trans masculine,Genderqueer 0.00170
## gender_identity_factorOther (Please specify) 0.00150
## gender_identity_factorMale,Transgender,Female to male transgender/FTM,Trans male/Trans masculine 0.00000
## gender_identity_factorGenderqueer,Nonbinary 0.00180
## gender_identity_factorFemale,Genderqueer,Not sure 0.00190
## gender_identity_factorMale,Female to male transgender/FTM,Trans male/Trans masculine 0.00150
## gender_identity_factorGenderqueer,Gender expansive,Agender,Other (Please specify) 0.00140
## gender_identity_factorFemale 0.00230
## gender_identity_factorTransgender,Genderqueer 0.00000
## gender_identity_factorFemale,Androgynous 0.00000
## gender_identity_factorMale to female transgender/MTF,Trans female/Trans feminine 0.00000
## gender_identity_factorNonbinary 0.00060
##
##
## Prediction Metrics:
## Deviance Explained S.E. Min Max
## Train Sample 0.012 0.002 0.007 0.014
## CV-Tune Holdout 0.005 0.003 0.003 0.006
## CV-Test Holdout 0.005 0.003 -0.001 0.007
## =======================================================

toc()

## 274.765 sec elapsed

## For BA-SSI

yes_data_pre_post_recoded_youth_only_ba_ssi_selection <- yes_data_pre_post_recoded_youth_only %>%
 dplyr::select(ssi_choice_factor, age_range_factor, bio_sex_factor, gender_identity_factor, b_mfq_mean) %>% na.omit() %>%
 mutate(ssi_choice_character = as.character(ssi_choice_factor),
 ba_ssi_choice_character = case_when(
 ssi_choice_character == "Project Personality" ~ "No",
 ssi_choice_character == "BA-SSI" ~ "Yes",
 ssi_choice_character == "Self-Hate SSI" ~ "No"),
 ba_ssi_choice_factor = as.factor(ba_ssi_choice_character)) %>%
 dplyr::select(-ssi_choice_factor,-ssi_choice_character,-ba_ssi_choice_character)

predict_ba_ssi_glm <- glm(ba_ssi_choice_factor ~ ., data = yes_data_pre_post_recoded_youth_only_ba_ssi_selection, family = "binomial")

## Way too colinear to run these in a traditional logistic regression

check_collinearity(predict_ba_ssi_glm)

## # Check for Multicollinearity

## Have to deviate and use a method that's more robust to collinearity

## Let's switch to an elastic net with a binomial outcome/nested cross-validation
tic()
predict_ba_ssi_choice_el_net <- beset_elnet(ba_ssi_choice_factor ~ ., data = yes_data_pre_post_recoded_youth_only_ba_ssi_selection, family = "binomial", seed = 33, nest_cv = T)
predict_ba_ssi_choice_el_net

##
## Results of nested 10-fold cross-validation repeated 10 times
## =======================================================
## Most conservative tuning parameters within
## 1 SE of best cross-validation Mean Cross Entropy:
## Mean S.E. Min Max
## alpha 0.897 0.082 0.745 0.990
## lambda 0.140 0.182 0.025 0.719
##
##
## Non-zero coefficients ranked in order of importance:
## Stnd.Coef.
## b_mfq_mean 0.06310
## gender_identity_factorGenderqueer 0.05410
## gender_identity_factorFemale 0.03340
## gender_identity_factorOther (Please specify) -0.02590
## gender_identity_factorTransgender,Genderqueer 0.01460
## gender_identity_factorFemale,Female to male transgender/FTM 0.01080
## gender_identity_factorFemale,Nonbinary 0.01070
## gender_identity_factorMale,Transgender,Female to male transgender/FTM 0.01070
## gender_identity_factorMale,Female,Nonbinary 0.01050
## gender_identity_factorFemale,Androgynous -0.01040
## gender_identity_factorTransgender,Nonbinary 0.01030
## gender_identity_factorMale,Transgender,Female to male transgender/FTM,Trans male/Trans masculine 0.00990
## gender_identity_factorMale,Female,Genderqueer,Androgynous,Nonbinary,Agender 0.00980
## gender_identity_factorMale,Intersex 0.00940
## gender_identity_factorNot sure -0.00890
## bio_sex_factorIntersex 0.00780
## bio_sex_factorMale -0.00450
## gender_identity_factorMale,Female to male transgender/FTM -0.00410
## gender_identity_factorFemale,Not sure -0.00320
## gender_identity_factorNonbinary -0.00260
## age_range_factor10 or younger 0.00250
## gender_identity_factorMale,Female,Transgender,Female to male transgender/FTM,Male to female transgender/MTF,Trans male/Trans masculine,Trans female/Trans feminine,Genderqueer,Gender expansive,Intersex,Androgynous,Nonbinary,Two-spirited,Third gender,Agender,Not sure,Other (Please specify) 0.00220
## gender_identity_factorFemale,Genderqueer,Not sure -0.00140
## gender_identity_factorFemale,Transgender -0.00120
## gender_identity_factorFemale,Androgynous,Two-spirited -0.00120
## age_range_factor14 to 16 0.00120
## gender_identity_factorAndrogynous,Third gender -0.00120
## gender_identity_factorGenderqueer,Nonbinary -0.00110
## gender_identity_factorMale,Transgender,Female to male transgender/FTM,Trans male/Trans masculine,Genderqueer -0.00100
## gender_identity_factorMale,Female,Transgender -0.00100
## gender_identity_factorMale,Female to male transgender/FTM,Trans male/Trans masculine -0.00100
## gender_identity_factorMale,Not sure -0.00100
## gender_identity_factorGender expansive -0.00090
## gender_identity_factorGenderqueer,Gender expansive,Agender,Other (Please specify) -0.00080
## age_range_factor17 or older -0.00070
## gender_identity_factorMale -0.00060
## gender_identity_factorMale to female transgender/MTF,Trans female/Trans feminine -0.00050
## S.E.
## b_mfq_mean 0.00746
## gender_identity_factorGenderqueer 0.00819
## gender_identity_factorFemale 0.00746
## gender_identity_factorOther (Please specify) 0.00536
## gender_identity_factorTransgender,Genderqueer 0.00431
## gender_identity_factorFemale,Female to male transgender/FTM 0.00350
## gender_identity_factorFemale,Nonbinary 0.00351
## gender_identity_factorMale,Transgender,Female to male transgender/FTM 0.00358
## gender_identity_factorMale,Female,Nonbinary 0.00350
## gender_identity_factorFemale,Androgynous 0.00375
## gender_identity_factorTransgender,Nonbinary 0.00342
## gender_identity_factorMale,Transgender,Female to male transgender/FTM,Trans male/Trans masculine 0.00432
## gender_identity_factorMale,Female,Genderqueer,Androgynous,Nonbinary,Agender 0.00342
## gender_identity_factorMale,Intersex 0.00326
## gender_identity_factorNot sure 0.00417
## bio_sex_factorIntersex 0.00271
## bio_sex_factorMale 0.00393
## gender_identity_factorMale,Female to male transgender/FTM 0.00218
## gender_identity_factorFemale,Not sure 0.00193
## gender_identity_factorNonbinary 0.00265
## age_range_factor10 or younger 0.00232
## gender_identity_factorMale,Female,Transgender,Female to male transgender/FTM,Male to female transgender/MTF,Trans male/Trans masculine,Trans female/Trans feminine,Genderqueer,Gender expansive,Intersex,Androgynous,Nonbinary,Two-spirited,Third gender,Agender,Not sure,Other (Please specify) 0.00148
## gender_identity_factorFemale,Genderqueer,Not sure 0.00110
## gender_identity_factorFemale,Transgender 0.00105
## gender_identity_factorFemale,Androgynous,Two-spirited 0.00104
## age_range_factor14 to 16 0.00130
## gender_identity_factorAndrogynous,Third gender 0.00104
## gender_identity_factorGenderqueer,Nonbinary 0.00105
## gender_identity_factorMale,Transgender,Female to male transgender/FTM,Trans male/Trans masculine,Genderqueer 0.00098
## gender_identity_factorMale,Female,Transgender 0.00091
## gender_identity_factorMale,Female to male transgender/FTM,Trans male/Trans masculine 0.00093
## gender_identity_factorMale,Not sure 0.00091
## gender_identity_factorGender expansive 0.00088
## gender_identity_factorGenderqueer,Gender expansive,Agender,Other (Please specify) 0.00084
## age_range_factor17 or older 0.00096
## gender_identity_factorMale 0.00088
## gender_identity_factorMale to female transgender/MTF,Trans female/Trans feminine 0.00070
## Min
## b_mfq_mean 0.05520
## gender_identity_factorGenderqueer 0.04280
## gender_identity_factorFemale 0.02680
## gender_identity_factorOther (Please specify) -0.03170
## gender_identity_factorTransgender,Genderqueer 0.01050
## gender_identity_factorFemale,Female to male transgender/FTM 0.00660
## gender_identity_factorFemale,Nonbinary 0.00630
## gender_identity_factorMale,Transgender,Female to male transgender/FTM 0.00620
## gender_identity_factorMale,Female,Nonbinary 0.00690
## gender_identity_factorFemale,Androgynous -0.01680
## gender_identity_factorTransgender,Nonbinary 0.00640
## gender_identity_factorMale,Transgender,Female to male transgender/FTM,Trans male/Trans masculine 0.00670
## gender_identity_factorMale,Female,Genderqueer,Androgynous,Nonbinary,Agender 0.00610
## gender_identity_factorMale,Intersex 0.00350
## gender_identity_factorNot sure -0.01180
## bio_sex_factorIntersex 0.00510
## bio_sex_factorMale -0.00850
## gender_identity_factorMale,Female to male transgender/FTM -0.00860
## gender_identity_factorFemale,Not sure -0.00790
## gender_identity_factorNonbinary -0.00650
## age_range_factor10 or younger 0.00000
## gender_identity_factorMale,Female,Transgender,Female to male transgender/FTM,Male to female transgender/MTF,Trans male/Trans masculine,Trans female/Trans feminine,Genderqueer,Gender expansive,Intersex,Androgynous,Nonbinary,Two-spirited,Third gender,Agender,Not sure,Other (Please specify) 0.00040
## gender_identity_factorFemale,Genderqueer,Not sure -0.00430
## gender_identity_factorFemale,Transgender -0.00430
## gender_identity_factorFemale,Androgynous,Two-spirited -0.00400
## age_range_factor14 to 16 0.00000
## gender_identity_factorAndrogynous,Third gender -0.00430
## gender_identity_factorGenderqueer,Nonbinary -0.00350
## gender_identity_factorMale,Transgender,Female to male transgender/FTM,Trans male/Trans masculine,Genderqueer -0.00320
## gender_identity_factorMale,Female,Transgender -0.00280
## gender_identity_factorMale,Female to male transgender/FTM,Trans male/Trans masculine -0.00350
## gender_identity_factorMale,Not sure -0.00280
## gender_identity_factorGender expansive -0.00280
## gender_identity_factorGenderqueer,Gender expansive,Agender,Other (Please specify) -0.00300
## age_range_factor17 or older -0.00180
## gender_identity_factorMale -0.00270
## gender_identity_factorMale to female transgender/MTF,Trans female/Trans feminine -0.00250
## Max
## b_mfq_mean 0.07060
## gender_identity_factorGenderqueer 0.06040
## gender_identity_factorFemale 0.03970
## gender_identity_factorOther (Please specify) -0.02200
## gender_identity_factorTransgender,Genderqueer 0.01890
## gender_identity_factorFemale,Female to male transgender/FTM 0.01470
## gender_identity_factorFemale,Nonbinary 0.01550
## gender_identity_factorMale,Transgender,Female to male transgender/FTM 0.01500
## gender_identity_factorMale,Female,Nonbinary 0.01590
## gender_identity_factorFemale,Androgynous -0.00670
## gender_identity_factorTransgender,Nonbinary 0.01520
## gender_identity_factorMale,Transgender,Female to male transgender/FTM,Trans male/Trans masculine 0.01480
## gender_identity_factorMale,Female,Genderqueer,Androgynous,Nonbinary,Agender 0.01390
## gender_identity_factorMale,Intersex 0.01420
## gender_identity_factorNot sure -0.00430
## bio_sex_factorIntersex 0.01010
## bio_sex_factorMale -0.00010
## gender_identity_factorMale,Female to male transgender/FTM -0.00080
## gender_identity_factorFemale,Not sure -0.00040
## gender_identity_factorNonbinary 0.00000
## age_range_factor10 or younger 0.00640
## gender_identity_factorMale,Female,Transgender,Female to male transgender/FTM,Male to female transgender/MTF,Trans male/Trans masculine,Trans female/Trans feminine,Genderqueer,Gender expansive,Intersex,Androgynous,Nonbinary,Two-spirited,Third gender,Agender,Not sure,Other (Please specify) 0.00410
## gender_identity_factorFemale,Genderqueer,Not sure 0.00000
## gender_identity_factorFemale,Transgender 0.00000
## gender_identity_factorFemale,Androgynous,Two-spirited 0.00000
## age_range_factor14 to 16 0.00390
## gender_identity_factorAndrogynous,Third gender 0.00000
## gender_identity_factorGenderqueer,Nonbinary 0.00000
## gender_identity_factorMale,Transgender,Female to male transgender/FTM,Trans male/Trans masculine,Genderqueer 0.00000
## gender_identity_factorMale,Female,Transgender 0.00000
## gender_identity_factorMale,Female to male transgender/FTM,Trans male/Trans masculine 0.00000
## gender_identity_factorMale,Not sure 0.00000
## gender_identity_factorGender expansive 0.00000
## gender_identity_factorGenderqueer,Gender expansive,Agender,Other (Please specify) 0.00000
## age_range_factor17 or older 0.00000
## gender_identity_factorMale 0.00000
## gender_identity_factorMale to female transgender/MTF,Trans female/Trans feminine 0.00000
##
##
## Prediction Metrics:
## Deviance Explained S.E. Min Max
## Train Sample 0.024 0.005 0.017 0.030
## CV-Tune Holdout 0.005 0.005 0.004 0.007
## CV-Test Holdout 0.003 0.005 -0.002 0.008
## =======================================================

toc()

## 295.025 sec elapsed

## For Project Care

yes_data_pre_post_recoded_youth_only_project_care_selection <- yes_data_pre_post_recoded_youth_only %>%
 dplyr::select(ssi_choice_factor, age_range_factor, bio_sex_factor, gender_identity_factor, b_mfq_mean) %>% na.omit() %>%
 mutate(ssi_choice_character = as.character(ssi_choice_factor),
 project_care_ssi_choice_character = case_when(
 ssi_choice_character == "Project Personality" ~ "No",
 ssi_choice_character == "BA-SSI" ~ "No",
 ssi_choice_character == "Self-Hate SSI" ~ "Yes"),
 project_care_ssi_choice_factor = as.factor(project_care_ssi_choice_character)) %>%
 dplyr::select(-ssi_choice_factor,-ssi_choice_character,-project_care_ssi_choice_character)

predict_project_care_glm <- glm(project_care_ssi_choice_factor ~ ., data = yes_data_pre_post_recoded_youth_only_project_care_selection, family = "binomial")

## Way too colinear to run these in a traditional logistic regression

check_collinearity(predict_project_care_glm)

## # Check for Multicollinearity

## Have to deviate and use a method that's more robust to collinearity

## Let's switch to an elastic net with a binomial outcome/nested cross-validation
tic()
predict_project_care_choice_el_net <- beset_elnet(project_care_ssi_choice_factor ~ ., data = yes_data_pre_post_recoded_youth_only_project_care_selection, family = "binomial", seed = 33, nest_cv = T)
predict_project_care_choice_el_net

##
## Results of nested 10-fold cross-validation repeated 10 times
## =======================================================
## Most conservative tuning parameters within
## 1 SE of best cross-validation Mean Cross Entropy:
## Mean S.E. Min Max
## alpha 0.966 0.046 0.892 0.990
## lambda 0.058 0.048 0.026 0.162
##
##
## Non-zero coefficients ranked in order of importance:
## Stnd.Coef.
## gender_identity_factorFemale,Not sure 0.05310
## gender_identity_factorMale 0.03230
## gender_identity_factorFemale,Androgynous 0.02910
## gender_identity_factorMale,Female,Transgender 0.02110
## gender_identity_factorAndrogynous,Third gender 0.02090
## gender_identity_factorGender expansive 0.02000
## gender_identity_factorMale to female transgender/MTF,Trans female/Trans feminine 0.01920
## bio_sex_factorMale 0.01590
## age_range_factor10 or younger 0.00860
## gender_identity_factorNot sure -0.00610
## gender_identity_factorOther (Please specify) 0.00420
## gender_identity_factorFemale -0.00080
## age_range_factor17 or older 0.00060
## gender_identity_factorGenderqueer -0.00020
## gender_identity_factorMale,Transgender,Female to male transgender/FTM,Trans male/Trans masculine -0.00010
## age_range_factor14 to 16 -0.00010
## gender_identity_factorMale,Female to male transgender/FTM -0.00010
## S.E.
## gender_identity_factorFemale,Not sure 0.01601
## gender_identity_factorMale 0.01334
## gender_identity_factorFemale,Androgynous 0.01096
## gender_identity_factorMale,Female,Transgender 0.00723
## gender_identity_factorAndrogynous,Third gender 0.00732
## gender_identity_factorGender expansive 0.00697
## gender_identity_factorMale to female transgender/MTF,Trans female/Trans feminine 0.00699
## bio_sex_factorMale 0.00985
## age_range_factor10 or younger 0.00491
## gender_identity_factorNot sure 0.00357
## gender_identity_factorOther (Please specify) 0.00296
## gender_identity_factorFemale 0.00168
## age_range_factor17 or older 0.00101
## gender_identity_factorGenderqueer 0.00037
## gender_identity_factorMale,Transgender,Female to male transgender/FTM,Trans male/Trans masculine 0.00035
## age_range_factor14 to 16 0.00031
## gender_identity_factorMale,Female to male transgender/FTM 0.00016
## Min
## gender_identity_factorFemale,Not sure 0.03250
## gender_identity_factorMale 0.02380
## gender_identity_factorFemale,Androgynous 0.01090
## gender_identity_factorMale,Female,Transgender 0.01220
## gender_identity_factorAndrogynous,Third gender 0.01220
## gender_identity_factorGender expansive 0.01220
## gender_identity_factorMale to female transgender/MTF,Trans female/Trans feminine 0.00810
## bio_sex_factorMale 0.00040
## age_range_factor10 or younger 0.00030
## gender_identity_factorNot sure -0.01440
## gender_identity_factorOther (Please specify) 0.00000
## gender_identity_factorFemale -0.00420
## age_range_factor17 or older 0.00000
## gender_identity_factorGenderqueer -0.00110
## gender_identity_factorMale,Transgender,Female to male transgender/FTM,Trans male/Trans masculine -0.00110
## age_range_factor14 to 16 -0.00100
## gender_identity_factorMale,Female to male transgender/FTM -0.00050
## Max
## gender_identity_factorFemale,Not sure 0.08430
## gender_identity_factorMale 0.05120
## gender_identity_factorFemale,Androgynous 0.04210
## gender_identity_factorMale,Female,Transgender 0.03640
## gender_identity_factorAndrogynous,Third gender 0.03620
## gender_identity_factorGender expansive 0.03230
## gender_identity_factorMale to female transgender/MTF,Trans female/Trans feminine 0.03340
## bio_sex_factorMale 0.02940
## age_range_factor10 or younger 0.01290
## gender_identity_factorNot sure -0.00060
## gender_identity_factorOther (Please specify) 0.00880
## gender_identity_factorFemale 0.00000
## age_range_factor17 or older 0.00400
## gender_identity_factorGenderqueer 0.00000
## gender_identity_factorMale,Transgender,Female to male transgender/FTM,Trans male/Trans masculine 0.00000
## age_range_factor14 to 16 0.00000
## gender_identity_factorMale,Female to male transgender/FTM 0.00000
##
##
## Prediction Metrics:
## Deviance Explained S.E. Min Max
## Train Sample 0.018 0.006 0.009 0.033
## CV-Tune Holdout 0.003 0.004 0.001 0.005
## CV-Test Holdout -0.002 0.004 -0.005 0.002
## =======================================================

beep(sound = 3)
toc()

## 336.489 sec elapsed

### **Predicting Who Completed Their SSI and Who Did Not**

Using an elastic net, we only predict 0.1% of the variance in who completes their SSI with pre-registered predictors (age range, biological sex, gender identity, and baseline MFQ depression sum score).

yes_data_pre_post_recoded_youth_only_ssi_completion <- yes_data_pre_post_recoded_youth_only %>%
 dplyr::select(completed_ssi, age_range_factor, bio_sex_factor, gender_identity_factor, b_mfq_mean) %>% na.omit()

predict_ssi_completion_glm <- glm(completed_ssi ~ ., data = yes_data_pre_post_recoded_youth_only_ssi_completion, family = "binomial")

## Way too colinear to run these in a traditional logistic regression

check_collinearity(predict_ssi_completion_glm)

## # Check for Multicollinearity

## Have to deviate and use a method that's more robust to collinearity

## Let's switch to an elastic net with a binomial outcome/nested cross-validation
tic()
predict_ssi_completion_el_net <- beset_elnet(completed_ssi ~ ., data = yes_data_pre_post_recoded_youth_only_ssi_completion, family = "binomial", seed = 33, nest_cv = T)
predict_ssi_completion_el_net

##
## Results of nested 10-fold cross-validation repeated 10 times
## =======================================================
## Most conservative tuning parameters within
## 1 SE of best cross-validation Mean Cross Entropy:
## Mean S.E. Min Max
## alpha 0.990 0.000 0.990 0.990
## lambda 0.039 0.004 0.034 0.045
##
##
## Non-zero coefficients ranked in order of importance:
## Stnd.Coef.
## age_range_factor14 to 16 -0.09800
## age_range_factor17 or older -0.03480
## gender_identity_factorMale,Female to male transgender/FTM 0.02910
## gender_identity_factorNonbinary 0.01050
## age_range_factor10 or younger 0.01000
## gender_identity_factorMale,Female,Nonbinary 0.00610
## gender_identity_factorMale,Transgender,Female to male transgender/FTM 0.00590
## gender_identity_factorMale,Female to male transgender/FTM,Trans male/Trans masculine 0.00580
## gender_identity_factorMale,Female,Genderqueer,Androgynous,Nonbinary,Agender 0.00570
## gender_identity_factorMale,Female,Transgender 0.00520
## gender_identity_factorGenderqueer,Gender expansive,Agender,Other (Please specify) 0.00310
## gender_identity_factorGender expansive 0.00080
## gender_identity_factorMale to female transgender/MTF,Trans female/Trans feminine 0.00070
## b_mfq_mean -0.00050
## gender_identity_factorNot sure -0.00040
## gender_identity_factorOther (Please specify) -0.00020
## gender_identity_factorMale,Transgender,Female to male transgender/FTM,Trans male/Trans masculine 0.00000
## gender_identity_factorFemale,Androgynous 0.00000
## S.E.
## age_range_factor14 to 16 0.02410
## age_range_factor17 or older 0.01485
## gender_identity_factorMale,Female to male transgender/FTM 0.00981
## gender_identity_factorNonbinary 0.00608
## age_range_factor10 or younger 0.00610
## gender_identity_factorMale,Female,Nonbinary 0.00266
## gender_identity_factorMale,Transgender,Female to male transgender/FTM 0.00265
## gender_identity_factorMale,Female to male transgender/FTM,Trans male/Trans masculine 0.00265
## gender_identity_factorMale,Female,Genderqueer,Androgynous,Nonbinary,Agender 0.00263
## gender_identity_factorMale,Female,Transgender 0.00250
## gender_identity_factorGenderqueer,Gender expansive,Agender,Other (Please specify) 0.00183
## gender_identity_factorGender expansive 0.00066
## gender_identity_factorMale to female transgender/MTF,Trans female/Trans feminine 0.00063
## b_mfq_mean 0.00080
## gender_identity_factorNot sure 0.00114
## gender_identity_factorOther (Please specify) 0.00053
## gender_identity_factorMale,Transgender,Female to male transgender/FTM,Trans male/Trans masculine 0.00013
## gender_identity_factorFemale,Androgynous 0.00006
## Min
## age_range_factor14 to 16 -0.14630
## age_range_factor17 or older -0.06930
## gender_identity_factorMale,Female to male transgender/FTM 0.01530
## gender_identity_factorNonbinary 0.00190
## age_range_factor10 or younger 0.00240
## gender_identity_factorMale,Female,Nonbinary 0.00120
## gender_identity_factorMale,Transgender,Female to male transgender/FTM 0.00100
## gender_identity_factorMale,Female to male transgender/FTM,Trans male/Trans masculine 0.00160
## gender_identity_factorMale,Female,Genderqueer,Androgynous,Nonbinary,Agender 0.00100
## gender_identity_factorMale,Female,Transgender 0.00160
## gender_identity_factorGenderqueer,Gender expansive,Agender,Other (Please specify) 0.00000
## gender_identity_factorGender expansive 0.00000
## gender_identity_factorMale to female transgender/MTF,Trans female/Trans feminine 0.00000
## b_mfq_mean -0.00180
## gender_identity_factorNot sure -0.00360
## gender_identity_factorOther (Please specify) -0.00170
## gender_identity_factorMale,Transgender,Female to male transgender/FTM,Trans male/Trans masculine 0.00000
## gender_identity_factorFemale,Androgynous 0.00000
## Max
## age_range_factor14 to 16 -0.05980
## age_range_factor17 or older -0.01320
## gender_identity_factorMale,Female to male transgender/FTM 0.04510
## gender_identity_factorNonbinary 0.01900
## age_range_factor10 or younger 0.01380
## gender_identity_factorMale,Female,Nonbinary 0.01070
## gender_identity_factorMale,Transgender,Female to male transgender/FTM 0.01070
## gender_identity_factorMale,Female to male transgender/FTM,Trans male/Trans masculine 0.00880
## gender_identity_factorMale,Female,Genderqueer,Androgynous,Nonbinary,Agender 0.01070
## gender_identity_factorMale,Female,Transgender 0.00860
## gender_identity_factorGenderqueer,Gender expansive,Agender,Other (Please specify) 0.00670
## gender_identity_factorGender expansive 0.00180
## gender_identity_factorMale to female transgender/MTF,Trans female/Trans feminine 0.00170
## b_mfq_mean 0.00000
## gender_identity_factorNot sure 0.00000
## gender_identity_factorOther (Please specify) 0.00000
## gender_identity_factorMale,Transgender,Female to male transgender/FTM,Trans male/Trans masculine 0.00040
## gender_identity_factorFemale,Androgynous 0.00020
##
##
## Prediction Metrics:
## Deviance Explained S.E. Min Max
## Train Sample 0.013 0.004 0.007 0.019
## CV-Tune Holdout 0.005 0.003 0.002 0.007
## CV-Test Holdout 0.001 0.004 -0.003 0.007
## =======================================================

beep(sound = 3)
toc()

## 309.902 sec elapsed

### **Predicting Mean Program Feedback Score**

Using an elastic net we only predict -0.3% of the variance in the Program Feedback Scale using pre-registered predictors (age range, biological sex, gender identity, and baseline MFQ depression sum score).

yes_data_pre_post_recoded_youth_only_pfs <- yes_data_pre_post_recoded_youth_only %>%
 dplyr::select(pi_pfs_mean, age_range_factor, bio_sex_factor, gender_identity_factor, b_mfq_mean) %>% na.omit()

predict_pfs_lm <- lm(pi_pfs_mean ~ ., data = yes_data_pre_post_recoded_youth_only_pfs)

## Way too colinear to run these in a traditional logistic regression

check_collinearity(predict_pfs_lm)

## # Check for Multicollinearity
##
## Low Correlation
##
## Parameter VIF Increased SE
## age_range_factor 1.41 1.19
## b_mfq_mean 1.31 1.15
##
## High Correlation
##
## Parameter VIF Increased SE
## bio_sex_factor 17.21 4.15
## gender_identity_factor 21.23 4.61

## Have to deviate and use a method that's more robust to collinearity

## Let's switch to an elastic net with a binomial outcome/nested cross-validation
tic()
predict_pfs_el_net <- beset_elnet(pi_pfs_mean ~ ., data = yes_data_pre_post_recoded_youth_only_pfs, seed = 33, nest_cv = T)
predict_pfs_el_net

##
## Results of nested 10-fold cross-validation repeated 10 times
## =======================================================
## Most conservative tuning parameters within
## 1 SE of best cross-validation Mean Squared Error:
## Mean S.E. Min Max
## alpha 0.990 0.000 0.990 0.99
## lambda 0.086 0.003 0.082 0.09
##
##
## No reliable predictors.
##
## Prediction Metrics:
## Variance Explained S.E. Min Max
## Train Sample 0.000 0.000 0.000 0.000
## CV-Tune Holdout -0.009 0.008 -0.011 -0.007
## CV-Test Holdout -0.003 0.006 -0.006 -0.001
## =======================================================

beep(sound = 3)
toc()

## 102.568 sec elapsed

**Supplemental Material: Shortening Self-Hate Scale**

**Table of Contents**

[Shortening the Self-Hate Scale 61](#_Toc37007956)

[The Self-Hate Scale Appears to Have 1 Factor In Adolescents 61](#_Toc37007957)

[What if We Reduce the Scale Down to the 3 Items That Load Most Strongly Onto the Latent Factor? 64](#_Toc37007958)

[How Correlated are The Short and Full Versions of the Self-Hate Scale? 65](#_Toc37007959)

[Do They Show Similar Correlations with Depression? 66](#_Toc37007960)

## **Shortening the Self-Hate Scale**

Here, we used confirmatory factor analysis (CFA) to formally select items for a shortened version of the Self-Hate Scale (Turnell et al., 2019) in an initial sample of adolescents (N = 246). After confirming the full Self-Hate scale loaded onto 1 factor, we then selected the three individual items with the highest factor loadings: “I hate myself” + “I feel disgusted when I think about myself” + “I feel ashamed of myself”. Next, we confirmed the shortened, 3-item scale was very highly correlated with the full 7-item scale (r = 0.97). Both the 3-item and full scale version of the Self-Hate Scale were highly correlated with a measure of depression, the Mood and Feelings Questionnaire (r’s = 0.76 and 0.80, respectively). Even after removing the “self-hate” item from the MFQ depression scale, it retained high correlations with both the 3-item and full scale versions of the Self-Hate Scale (r’s = 0.78 and 0.74, respectively). We therefore conclude we can interpret the 3-item version of the Self-Hate Scale in our Project YES adolescent sample.

### **The Self-Hate Scale Appears to Have 1 Factor In Adolescents**

The fit according to the CFA isn’t amazing, but I trust the parallel analysis saying one factor.

## How many factors does parallel analysis say we have? According to this, only 1
set.seed(33)
self_hate_parallel <- fa.parallel(self_hate_short_df_only,n.obs=246, fm = 'ml',
 fa = 'fa', n.iter = 50, quant = .95)

## Warning in fa.parallel(self_hate_short_df_only, n.obs = 246, fm = "ml", :
## You specified the number of subjects, implying a correlation matrix, but do
## not have a correlation matrix, correlations found


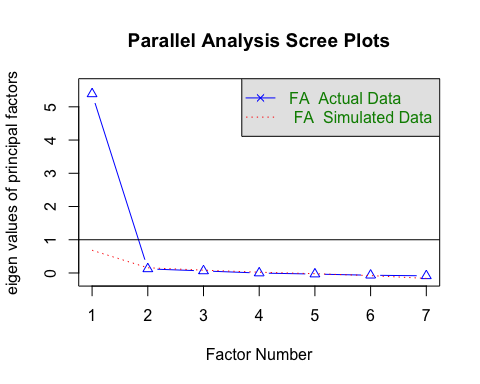


## Parallel analysis suggests that the number of factors = 1 and the number of components = NA

self_hate_parallel$nfact

## [1] 1

## Let's see what the highest loadings are

# specify the model
self_hate_cfa_model <- ' self hate =~ Q63_1 + Q63_2 + Q63_3 + Q63_4 + Q63_5 + Q63_6 + Q63_7 '

# fit the model
fit <- cfa(self_hate_cfa_model, data=self_hate_short_df_only)

# display summary output
summary(fit, fit.measures=TRUE)

## lavaan 0.6-5 ended normally after 26 iterations
##
## Estimator ML
## Optimization method NLMINB
## Number of free parameters 14
##
## Number of observations 246
##
## Model Test User Model:
##
## Test statistic 79.908
## Degrees of freedom 14
## P-value (Chi-square) 0.000
##
## Model Test Baseline Model:
##
## Test statistic 1873.683
## Degrees of freedom 21
## P-value 0.000
##
## User Model versus Baseline Model:
##
## Comparative Fit Index (CFI) 0.964
## Tucker-Lewis Index (TLI) 0.947
##
## Loglikelihood and Information Criteria:
##
## Loglikelihood user model (H0) -2676.020
## Loglikelihood unrestricted model (H1) -2636.066
##
## Akaike (AIC) 5380.040
## Bayesian (BIC) 5429.114
## Sample-size adjusted Bayesian (BIC) 5384.735
##
## Root Mean Square Error of Approximation:
##
## RMSEA 0.138
## 90 Percent confidence interval - lower 0.110
## 90 Percent confidence interval - upper 0.169
## P-value RMSEA <= 0.05 0.000
##
## Standardized Root Mean Square Residual:
##
## SRMR 0.024
##
## Parameter Estimates:
##
## Information Expected
## Information saturated (h1) model Structured
## Standard errors Standard
##
## Latent Variables:
## Estimate Std.Err z-value P(>|z|)
## selfhate =~
## Q63_1 1.000
## Q63_2 0.937 0.042 22.060 0.000
## Q63_3 1.023 0.042 24.424 0.000
## Q63_4 1.022 0.042 24.403 0.000
## Q63_5 0.971 0.046 21.126 0.000
## Q63_6 0.919 0.052 17.623 0.000
## Q63_7 0.893 0.047 19.059 0.000
##
## Variances:
## Estimate Std.Err z-value P(>|z|)
## .Q63_1 0.605 0.068 8.919 0.000
## .Q63_2 0.750 0.078 9.561 0.000
## .Q63_3 0.606 0.069 8.820 0.000
## .Q63_4 0.607 0.069 8.828 0.000
## .Q63_5 0.934 0.096 9.772 0.000
## .Q63_6 1.435 0.139 10.327 0.000
## .Q63_7 1.087 0.107 10.135 0.000
## selfhate 3.051 0.328 9.313 0.000

# get factor loadings
inspect(fit,what="std")$lambda

## selfht
## Q63_1 0.914
## Q63_2 0.884
## Q63_3 0.917
## Q63_4 0.917
## Q63_5 0.869
## Q63_6 0.801
## Q63_7 0.832

### **What if We Reduce the Scale Down to the 3 Items That Load Most Strongly Onto the Latent Factor?**

The items with the highest factor loadings in the CFA are Q63_1, Q63_3, Q63_4 in Project YES and correspond to “I hate myself” + “I feel disgusted when I think about myself” + “I feel ashamed of myself” which also happen to be face valid self-dislike/self-hatred terms. Running another parallel analysis tells us we also only have 1 factor using just these items.

## Creating a data frame with only the top 3 loading items
self_hate_short_df_only_3_item <- self_hate_short_df_only %>%
 dplyr::select(Q63_1, Q63_3, Q63_4)

## How many factors does parallel analysis say we have? According to this, only 1
set.seed(33)
self_hate_parallel_short <- fa.parallel(self_hate_short_df_only_3_item,n.obs=246, fm = 'ml',
 fa = 'fa', n.iter = 50, quant = .95)

## Warning in fa.parallel(self_hate_short_df_only_3_item, n.obs = 246, fm
## = "ml", : You specified the number of subjects, implying a correlation
## matrix, but do not have a correlation matrix, correlations found


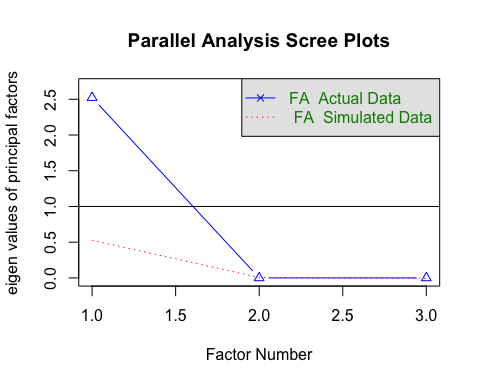


## Parallel analysis suggests that the number of factors = 1 and the number of components = NA

self_hate_parallel_short$nfact

## [1] 1

### **How Correlated are The Short and Full Versions of the Self-Hate Scale?**

The short (3 item) and full versions correlate with one another at r = 0.97

## Creating both versions + MFQ
self_hate_short_df <- self_hate_short_df %>%
 mutate(self_hate_long_mean = rowMeans(dplyr::select(self_hate_short_df, contains("Q63_")), na.rm = TRUE),
 self_hate_short_mean = rowMeans(dplyr::select(self_hate_short_df, Q63_1, Q63_3, Q63_4)),
 mfq_mean = rowMeans(dplyr::select(self_hate_short_df, contains("Q4_")), na.rm = TRUE),
 mfq_mean_no_self_dislike = rowMeans(dplyr::select(self_hate_short_df, contains("Q4_"), -Q4_8), na.rm = TRUE))

## Correlating the short/long versions
cor.test(self_hate_short_df$self_hate_long_mean, self_hate_short_df$self_hate_short_mean)

##
## Pearson's product-moment correlation
##
## data: self_hate_short_df$self_hate_long_mean and self_hate_short_df$self_hate_short_mean
## t = 66.806, df = 244, p-value < 2.2e-16
## alternative hypothesis: true correlation is not equal to 0
## 95 percent confidence interval:
## 0.9663542 0.9795162
## sample estimates:
## cor
## 0.9737367

### **Do They Show Similar Correlations with Depression?**

The full version correlates with the MFQ at r = 0.80(!!) and the short version correlates at r = 0.76(!!). These correlations are remarkably high, even when I take out the symptom that assesses self-hatred in the MFQ still see r = 0.78 for the full version and r = 0.74 for the short version. Overall the correlations for the long and short versions with depression definitely don’t differ from one another, so we can use the reduced scale in future versions of YES if we want to.

# Mean full self-hate scale & mean full MFQ
cor.test(self_hate_short_df$self_hate_long_mean, self_hate_short_df$mfq_mean)

##
## Pearson's product-moment correlation
##
## data: self_hate_short_df$self_hate_long_mean and self_hate_short_df$mfq_mean
## t = 21.005, df = 244, p-value < 2.2e-16
## alternative hypothesis: true correlation is not equal to 0
## 95 percent confidence interval:
## 0.7529268 0.8429103
## sample estimates:
## cor
## 0.8024341

# Mean short self-hate scale & mean full MFQ
cor.test(self_hate_short_df$self_hate_short_mean, self_hate_short_df$mfq_mean)

##
## Pearson's product-moment correlation
##
## data: self_hate_short_df$self_hate_short_mean and self_hate_short_df$mfq_mean
## t = 18.216, df = 244, p-value < 2.2e-16
## alternative hypothesis: true correlation is not equal to 0
## 95 percent confidence interval:
## 0.7005662 0.8075255
## sample estimates:
## cor
## 0.7591236

# Mean full self-hate scale & MFQ without the self-hatred item
cor.test(self_hate_short_df$self_hate_long_mean, self_hate_short_df$mfq_mean_no_self_dislike)

##
## Pearson's product-moment correlation
##
## data: self_hate_short_df$self_hate_long_mean and self_hate_short_df$mfq_mean_no_self_dislike
## t = 19.705, df = 244, p-value < 2.2e-16
## alternative hypothesis: true correlation is not equal to 0
## 95 percent confidence interval:
## 0.7301423 0.8276078
## sample estimates:
## cor
## 0.7836516

# Mean short self-hate scale & MFQ without the self-hatred item
cor.test(self_hate_short_df$self_hate_short_mean, self_hate_short_df$mfq_mean_no_self_dislike)

##
## Pearson's product-moment correlation
##
## data: self_hate_short_df$self_hate_short_mean and self_hate_short_df$mfq_mean_no_self_dislike
## t = 16.985, df = 244, p-value < 2.2e-16
## alternative hypothesis: true correlation is not equal to 0
## 95 percent confidence interval:
## 0.6729221 0.7885280
## sample estimates:
## cor
## 0.7360464
